# An Intravital Microscopy Toolbox to Study Mammary Gland Dynamics from Cellular Level to Organ Scale

**DOI:** 10.1007/s10911-021-09487-2

**Published:** 2021-05-04

**Authors:** Hendrik A. Messal, Jacco van Rheenen, Colinda L. G. J. Scheele

**Affiliations:** 1 Division of Molecular Pathology, Netherlands Cancer Institute, Oncode Institute, Plesmanlaan 121, 1066CX, Amsterdam, The Netherlands; 2 VIB-KULeuven Center for Cancer Biology, Herestraat 49, 3000 Leuven, Belgium

**Keywords:** Intravital microscopy, Mammary imaging window, Mammary gland development, Mammary gland homeostasis, Estrous cycle, Proliferative heterogeneity

## Abstract

The architecture of the mouse mammary gland is highly dynamic and constantly remodeled during pubertal development and estrous cycle-driven sprouting and regression of alveolar side branches. During each of these developmental stages, turnover is driven by distinct subsets of mammary epithelial cells. Extensive previous research has shed light on the unique morphological and cell biological characteristics of each stage. However, technological shortcomings failed to capture the dynamics and single-cell contributions to mammary remodeling. Here, we developed in vivo imaging strategies to follow the same mammary ducts over time and quantify the dynamics of mammary gland growth and remodeling from single-cell level to organ scale. Using a combination of intravital microscopy and genetic reporter systems we show how proliferative heterogeneity drives ductal morphogenesis during different developmental stages. To visualize pubertal growth at the cellular level, we performed long-term time-lapse imaging of extending terminal end buds through a mammary imaging window. We show that single-cells within the terminal end buds are extremely motile and continuously exchange position whilst the duct is elongating. To visualize short-term remodeling in the adult mammary gland at the single cell level, we performed multi-day intravital imaging in photoconvertible Kikume Green–Red mice and fluorescent ubiquitination-based cell cycle indicator mice. We demonstrate that the contribution of single-cells to estrous-driven remodeling is highly variable between cells in the same micro-environment. To assess the effects of this dynamic proliferative contribution on the long-term stability of tissue architecture, we developed a repeated skin flap method to assess mammary gland morphology by intravital microscopy over extended time spans for up to six months. Interestingly, in contrast to the short-term dynamic remodeling, the long-term morphology of the mammary gland remains remarkably stable. Together, our tool box of imaging strategies allows to identify and map transient and continuing dynamics of single cells to the architecture of the mammary gland.

**Supplementary Information:**

The online version contains supplementary material available at 10.1007/s10911-021-09487-2.

## Introduction

The mammary gland is a secretory organ that plays an essential role during the nursing of mammalian offspring, and is organized as a highly branched tree like structure that is embedded in the mammary fat pad. The mammary ductal tree consists of a bilayered epithelium with two major cell types; an outer layer of contractile myoepithelial or basal cells, and an inner layer of luminal cells [1]. The mammary duct is a remarkably dynamic tissue that undergoes various phases of growth and remodeling throughout life. Whilst the mammary anlagen are specified during embryonic development, the majority of ductal development occurs only during puberty, during which the full ductal tree is laid down through the process of branching morphogenesis [2, 3]. During adult life, the mammary gland undergoes rounds of remodeling driven by the hormonal cycle during which alveolar buds are formed and regress every 4 to 6 days [4]. Only when pregnancy occurs, the alveolar buds remain and further develop into mature alveoli, the milk producing units of the mammary gland. At the end of the lactation period, the process of involution takes place during which the developed alveoli undergo apoptosis and the mammary gland returns to a near pre-pregnancy stage.

The dynamic nature of the mammary epithelium makes a substantial proliferative demand on the mammary cells, suggesting the presence of actively dividing and long-lived mammary stem cells (MaSCs) throughout adult life. Interestingly, each developmental stage is characterized by proliferation at different anatomical locations and by different cell types and dynamics, suggesting the presence of developmental stage specific progenitor- or MaSC populations [1, 4]. Because of this developmental stage dependence, it is important to not rely on specific, pre-defined markers, but rather define stemness potential through functional experiments [2]. Previously, it was shown that during puberty, the pubertal progenitors are located in the terminal end buds, and these cells are the drivers of ductal elongation and bifurcation [2, 5–7]. Pubertal branching morphogenesis employs a constant rate of stochastic progenitor divisions over a few weeks, which ceases when the ducts reach a critical density [3, 8]. In contrast, adult remodeling involves successive rounds of proliferation and cell death that orchestrate the budding and regression of alveolar side branches throughout the entire mammary gland in the time scale of the estrous cycle of 4–6 days [9]. During adult stage, proliferation was shown to be scattered throughout the ductal epithelium [5, 10]. Although much is known about the molecular signaling pathways and hormones that drive ductal remodeling, the single cell dynamics during the distinct developmental processes remain largely unexplored [11–15]. To better understand the proliferative heterogeneity at these distinct anatomical locations during the different developmental phases, we developed a toolbox combining intravital microscopy (IVM) techniques with several reporter mouse models to follow proliferation and morphological changes within the mammary gland at different time scales, ranging from a few hours to several months [16]. By using IVM, we tracked the behavior and fate of mammary epithelial cells at the single cell level in three dimensions and over time. By combining IVM with several types of mammary imaging windows (MIW) we followed cell divisions, proliferative heterogeneity, and morphological changes over multiple days. Finally, by using a repeated skin flap approach, we assessed the long-term stability of the mammary gland over multiple weeks to months at the organ level. Together, these diverse IVM techniques provide a comprehensive toolbox to study mammary gland remodeling from the single cell level to the organ scale.

## Results

### Intravital Imaging Strategies to Visualize Short-term and Long-term Changes in the Pubertal Mammary Gland

**Fig. 1:**
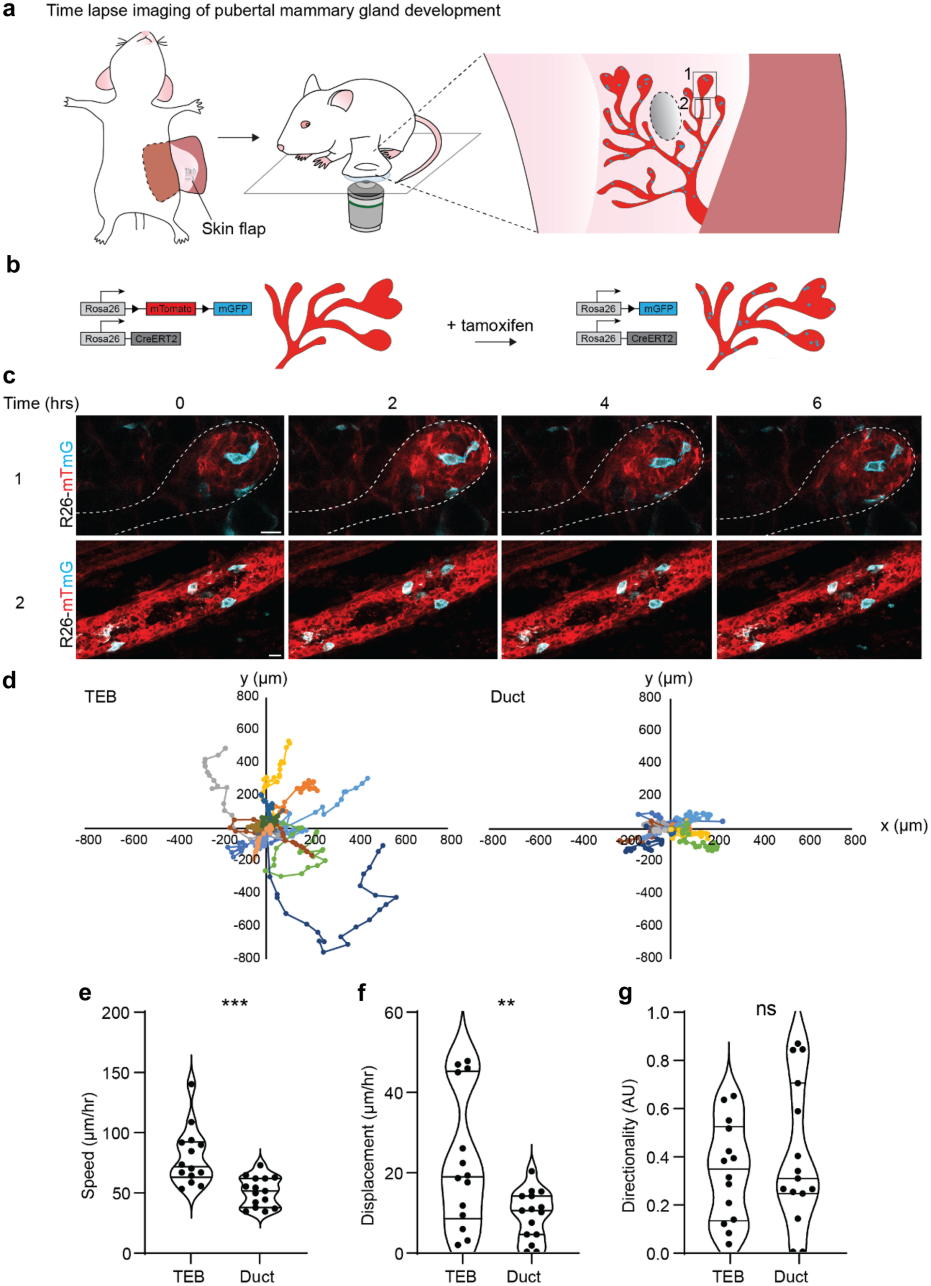
Time-lapse imaging of pubertal mammary gland development. **a** Cartoon of skin flap imaging workflow in the pubertal mammary gland. A skin flap surgery was performed on 5 week-old mice. Ducts and TEBs were followed over time for a minimum period of 8 h. **b** Schematic of the *R26-CreERT2;R26-mTmG* mouse model, before and after tamoxifen inducted Cre-recombination. **c** Representative confocal images (single Z-plane) of a TEB (upper panels) and the recently formed abutting duct (lower panels) in the *R26*-*mTmG* mouse model over time, demonstrating that the cells within the TEBs are highly motile, whereas the ductal cells are static. Recombined cells (mG) are depicted in cyan, non-recombined cells (mT) are depicted in red. Scale bars represent 10 µm. **d** Plot showing the migration tracks of 15 randomly picked TEB cells (left) and duct cells (right) over a period of 8 h. **e-g** Graph depicting average speed (µm/hour) **e**, average displacement (µm/hour) **f**, and directionality (AU) **g** of TEB cells and ductal cells over a period of 8 h. *n* = 15 cells tracked in 3 different mice. In each mouse, a position containing a TEB and the directly abutting duct was selected, and 5 randomly picked cells were tracked over the entire 8 h period within the TEB and the abutting duct. Lines in violin plots depict the median, and first and third quartile. P values are 0.0003 **e**, 0.007 **f**, and 0.4839 **g**, unpaired two-tailed t test. These data are representative of 6 independent experiments in 6 different mice. For each mouse approximately 10 positions (TEB, duct, or TEB with abutting duct) were selected and followed over a minimum time frame of 8 h. Each position was imaged in 4dimensions (xyzt)

Previously, we and others have shown that pubertal morphogenesis is solely driven by the TEB cells [2, 6]. These pubertal progenitors were shown to be transcriptionally and functionally heterogeneous, in which only the cells localized at the border of the TEB (but not at the tip) were contributing to ductal elongation in the short-term [2]. In the long-term however, all pubertal progenitors are contributing to ductal growth through cellular rearrangements within the TEBs, especially during bifurcation [2]. To further characterize pubertal pubertal progenitor rearrangements within the TEBs, we performed in vivo time-lapse imaging during pubertal development at 5 weeks-of-age using a skin flap (Fig. 1a, Table 1, and Supplementary Experimental Guide). To visualize the mammary epithelium, we made use of *R26-CreERt2;R26-mTmG* mice, in which all cells are labelled with membrane tdTomato (Fig. 1b, Table 2). We stochastically recombined the mTmG construct by administration of a low dose of tamoxifen (0.2 mg/25gram body weight), leading to a red-to-green switch in some cells throughout all tissues, including the mammary gland (Fig. 1b, and Table 2). Note that it is important to administer a low dose of tamoxifen, since higher doses were previously shown to delay or inhibit branching morphogenesis in the pubertal mammary gland [2, 17].

**Table 1:** IVM Techniques

Technique	Description	Overall duration	Duration per imaging session	Accessible area	Figures
*MIW*	Optical access via a surgically skin-implanted titanium ring with a coverglass insert (12 mm diameter).	days	Defined by the maximal duration of nonterminal anaesthesia (subject to institutional and national legislation)	All the ducts under the 12 mm cover glass	- Fig. 2, 5
*R.MIW*	Optical access via a surgically skin-implanted titanium ring with a replaceable coverglass insert (10 mm diameter).	days - weeks	Defined by the maximal duration of nonterminal anaesthesia (subject to institutional and national legislation)	All the ducts under the 10 mm cover glass	- Fig. 3
*Skin Flap*	Optical access via surgical exposure of the mammary gland.	months	~ 12 h. Longer exposure may compromise healthy tissue (tumours can be imaged for up to 40 h [28])	Up to ~20 mm^2	- terminal skin flap: Fig. 1, 4, - repeated skin flap: Fig. 6, 7

**Table 2:** Mouse Models

Name	Strain + Reference	Fluorophores	Resolution	Suitability		Figures
*R26-mTmG *x *R26-CreERT2*	B6.129(Cg)-*Gt(ROSA)26Sor tm4(ACTB-tdTomato,-EGFP)Luo */J 36271253340https://www.jax.org/strain/007676 https://onlinelibrary.wiley.com/doi/abs/10.1002/dvg.20335	constitutive membrane-tdTomato is switched to membrane-EGFP upon Cre-recombination	- single cells - ductal trees	- Local recombination allows the tracking of single cells - Strong fluorescence intensity allows imaging of deeper tissue layers	- Recombination of cells requires tamoxifen injection which may potentially intefere with cellular processes in the mammary gland	- single cell tracking: Fig. 1, 2, 3 - tracking of ductal trees: Fig. 7
*R26-FUCCI2*	RIKEN CLST CDB0203T 17830853340http://www2.clst.riken.jp/arg/reporter_mice.htmlhttps://dev.biologists.org/content/140/1/237	cell cycle driven expression of mCherryhCdt1(30/120) & mVenus-hGem(1/110)	- single cells	- Low mVenus intensity limits imaging to small ductal areas close to the tissue surface		- single cell tracking: Fig. 4
*CAG-KikGR-1*	RIKEN CLST CDB0201T-1 17830853340http://www2.clst.riken.jp/arg/reporter_mice.htmlhttps://science.sciencemag.org/content/316/5825/719	constitutive KikGR expression. Green to Red photoconversion with 405 nm light	- single cells - ductal segments	- Local photoconversion allows to follow the same groups of cells over ~7 days - Moderate fluorescence intensity allows to capture larger ductal segments		- single cell tracking: Fig. 5 - tracking of ductal segments: Fig. 5, 6; Suppl.Fig. 4

**Fig. 2:**
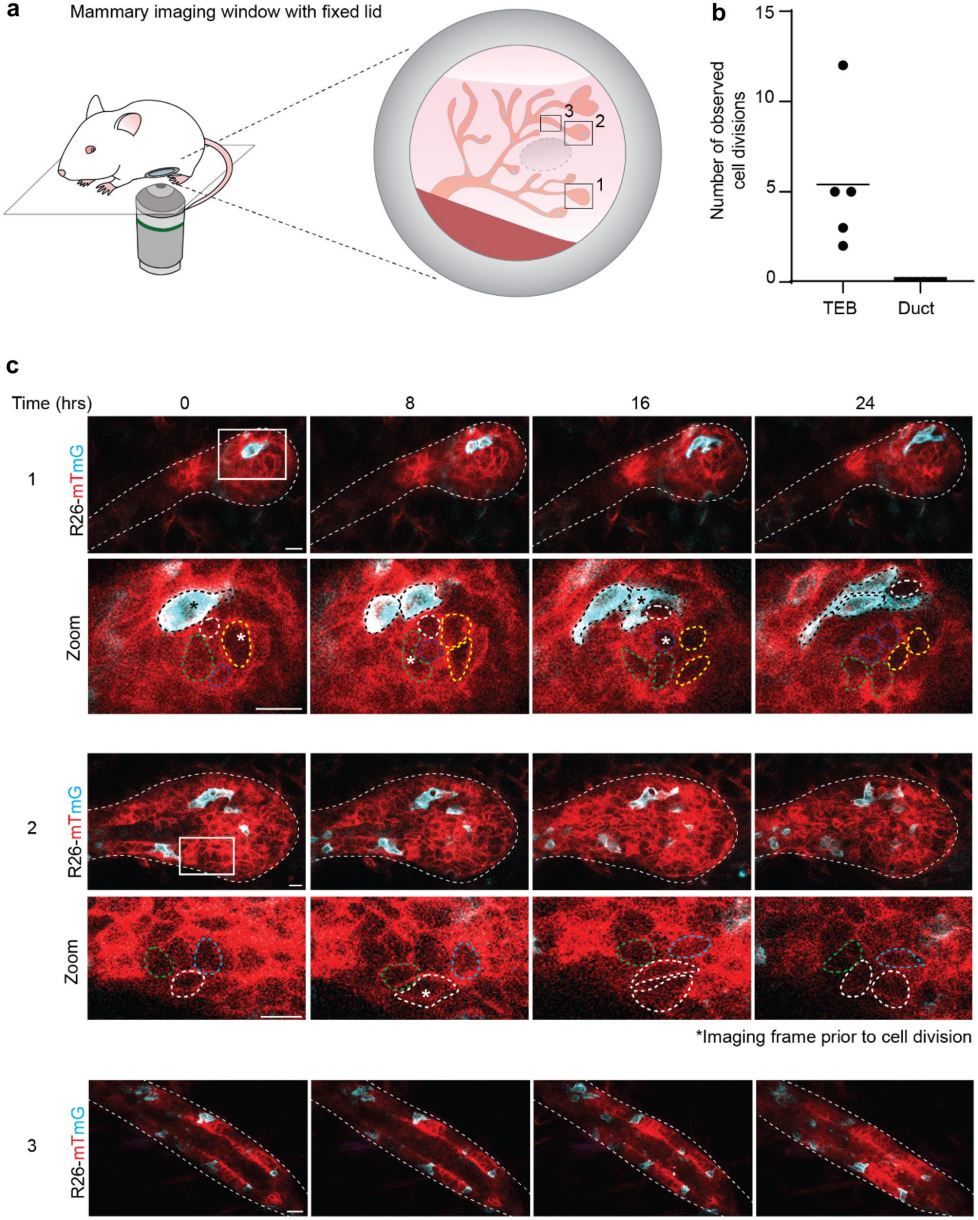
Long-term time-lapse imaging of pubertal mammary gland development. **a** Cartoon of MIW with fixed lid implanted on the pubertal mammary gland used for long-term time-lapse imaging. **b** Graph showing the number of observed cell divisions over 24 h in randomly picked TEBs and ducts. *n* = 5 TEBs and *n* = 7 ducts derived from 3 mice. **c** Representative confocal images (single Z-planes) of a TEB (panels 1 and 2) and recently formed duct (panels 3) in the *R26-CreERT2;R26-mTmG* mouse model followed over a period of 24 h. In the TEBs continuous cell divisions were observed (dividing cells are indicated with an *) whereas the ductal cells were static over time. Recombined cells (mT) are depicted in cyan, non-recombined cells (mG) are depicted in red. Scale bars represent 10 µm. These data are representative of 5 experiments in 5 different mice. In each mouse, the entire visible area through the MIW was imaged in 4 dimensions (xyzt), resulting in 3–5 positions per mouse that could be followed over 24 h

To study the dynamics of pubertal mammary gland development, we induced a red-to-green switch in *R26-CreERT2;R26-mTmG* mice at 3.5 weeks-of-age, which marks the onset of puberty. Around 5 weeks-of-age, we imaged TEBs and ducts of the developing mammary gland at cellular resolution for at least 8 h (Fig. 1c). In line with our previous results, we observed that TEB cells were exchanging their position over the course of the time-lapse imaging, whereas ductal cells were remarkably static (Fig. 1c and d). Although highly heterogeneous, on average both cell speed and cell displacement were significantly higher in the TEBs compared to the ducts (Fig. 1e and f, and Supplementary Fig. 1). Interestingly, pubertal progenitor movement did not show any directionality, indicating that the cell mixing within the TEB is stochastic (Fig. 1g). To visualize and quantify cellular proliferation within the developing mammary gland we adapted our imaging strategy for extended imaging times. To prevent dehydration of the mammary gland over longer time periods, we implanted a MIW with a fixed glass on top of the mammary fat pad (Fig. 2a, Table 1 and Supplementary Experimental Guide). By time-lapse imaging we followed the TEBs and ducts over a minimal period of 24 h to visualize cell division (Fig. 2b, c). In line with previous observations, cell divisions were only observed within the TEBs, whereas ducts were devoid of any proliferation (Fig. 2b, c).

**Fig. 3:**
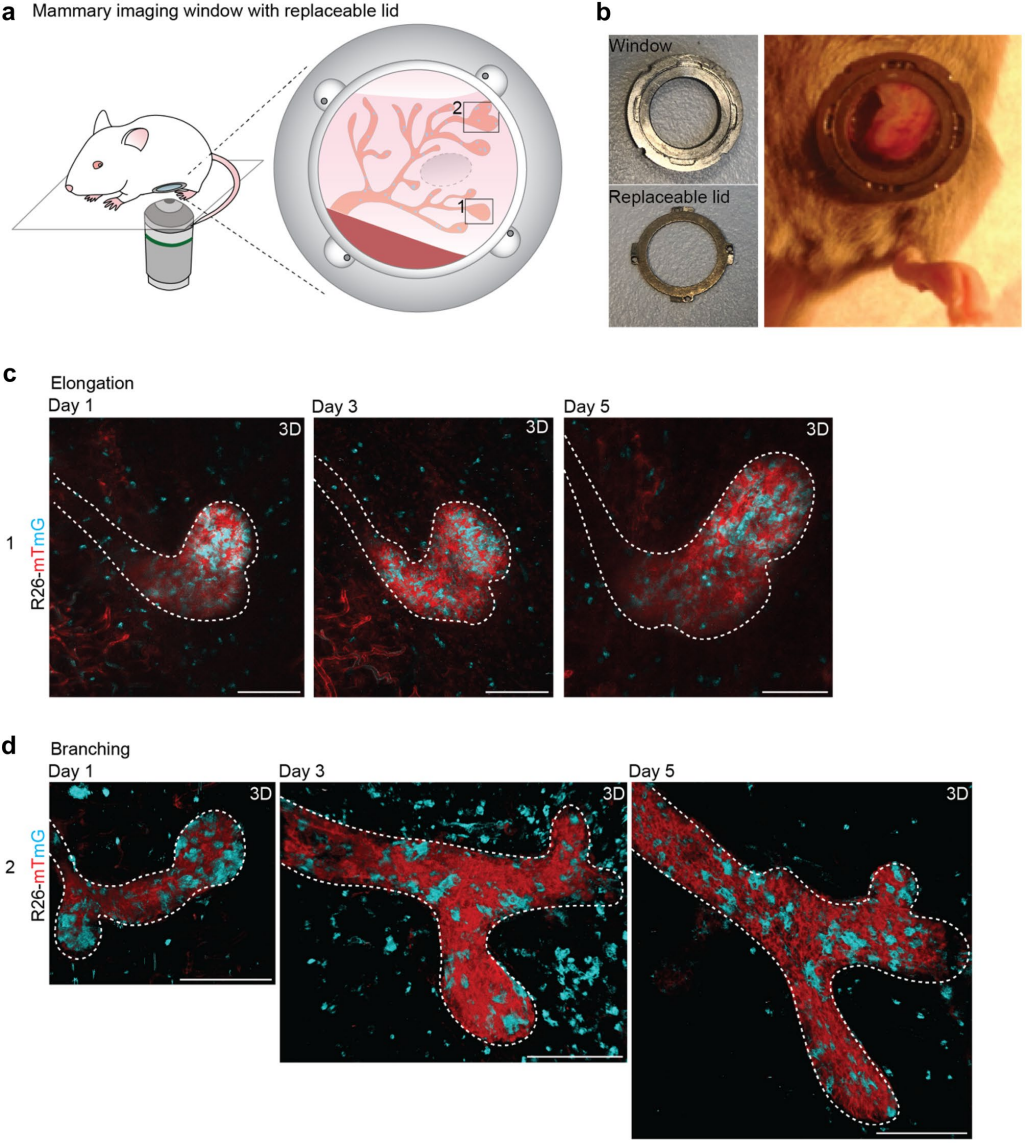
Multi-day imaging of pubertal mammary gland development. **a** Cartoon of the MIW with a replaceable lid implanted on the pubertal mammary gland used for multi-day imaging. **b** Picture of the MIW (left, top) with replaceable lid (left, bottom) implanted on top of the pubertal mammary gland (right picture). **c**-**d** Representative confocal images (3D rendering) of pubertal TEBs in an *R26-CreERT2;R26-mTmG* mouse showing growth, elongation **c**, and branching **d** over a period of 5 days. Scale bars represent 100 µm. These data are representative of 5 different experiments in 5 different mice. In each mouse, the entire visible area through the MIW with replaceable lid was imaged over multiple days in 3 dimensions (xyz), resulting in 3–5 positions per mouse that could be followed over multiple days

In order to understand how single cell dynamics result in morphological changes such as ductal elongation and branching events in the pubertal mammary gland, we adapted our imaging strategy towards a multi-day consecutive imaging strategy (Supplementary Experimental Guide). We performed repeated IVM imaging and revisited the same regions of interest over multiple days which enabled the visualization of morphological changes within the mammary gland (Fig. 3a). However, long-term visual access can sometimes be hampered by accumulation of protein and cellular debris at the coverglass [18]. Therefore, we redesigned the MIW with a replaceable lid (Fig. 3b, Table 1, Supplementary Fig. 2 and Supplementary Experimental Guide), which allows opening and cleaning of the glass (in a sterile environment) in between imaging sessions. This new design of the imaging window enabled the imaging of mammary ducts for several days at uncompromised visibility (Fig. 3c, d). Using this method, we followed the same TEBs over a period of 5 days and demonstrated both ductal elongation (Fig. 3c) and branching (Fig. 3d) at cellular resolution. The combination of stochastic cell tagging and intermittent IVM also informs on the dynamics of the ductal microenvironment. On some imaging days we observed an influx of stromal cells surrounding the elongating and branching ducts (Supplementary Fig. 3), which may be immune cells or fibroblasts involved in stromal remodeling to enable ductal elongation and branching.

Together, these IVM techniques allow to study pubertal branching morphogenesis at a cellular resolution over different time scales ranging from hours to days. With these methods we reveal the remarkable heterogeneity between TEB and ductal cell behavior during pubertal development.

## Proliferative Heterogeneity within the Adult Mammary Gland

After puberty, the proliferation in the TEBs ceases and TEBs fully regress to become bilayered ductal ends (also referred to as duct termini). Several studies have suggested that during adulthood proliferation is not focused within these ductal ends, but rather scattered throughout the epithelium [5, 10]. The main driver of adult mammary tissue turnover is the estrous cycle, which drives rounds of growth and regression within the murine mammary gland every 4–6 days under the influence of hormones estrogen and progesterone. Previously it was demonstrated that these hormones act on the hormone-receptor positive luminal cells, which in turn signal to the hormone receptor negative lineages to induce rounds of proliferation and regression through paracrine signaling [15]. Interestingly, although all mammary epithelial cells are able to proliferate in response to the hormonal signaling, only a subset of cells is responsive each individual cycle. Moreover, the extent of expansion is not the same in each estrous cycle, suggesting that there is a substantial proliferative heterogeneity within the mammary gland [5]. Although this proliferative heterogeneity has been extensively characterized ex vivo by FACS analysis [5, 19], the spatial organization of the response to the estrous cycle remains largely unknown. To better characterize the proliferative heterogeneity in vivo within the adult mammary tissue, we combined IVM with several proliferation-reporter mouse models.

**Fig. 4:**
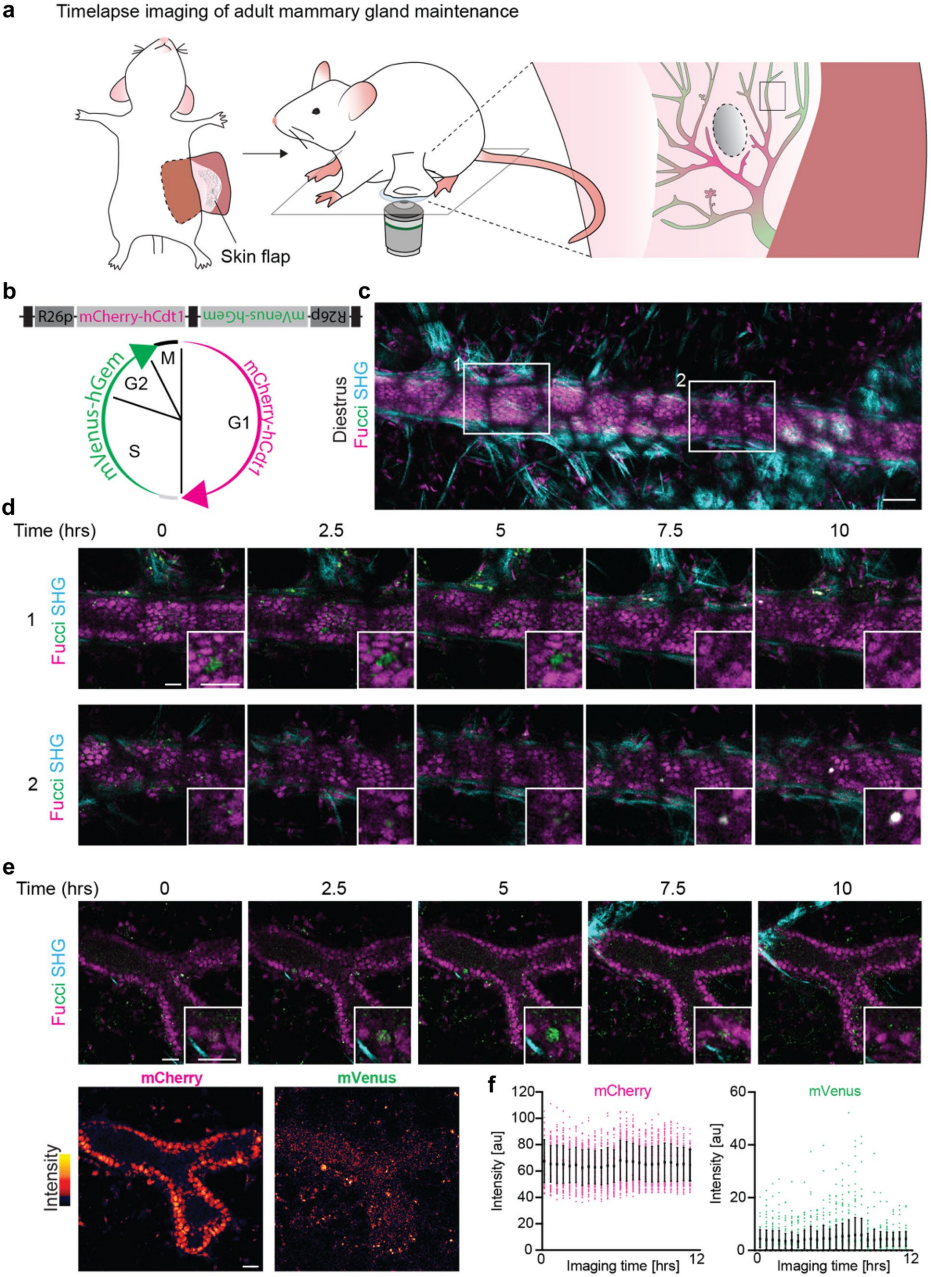
Time-lapse imaging of adult mammary gland maintenance. **a** Cartoon of adult mammary gland imaging through a terminal skin flap. **b** Cell cycle stage identification by nuclear mVenus (green) or mCherry (pink) expression in the *FUCCI2* mouse. The *FUCCI2* cassette consists of a bidirectionally conjugated mCherry-hCdt1 and mVenus-hGem under control of R26 promoters (R26p). Black boxes indicate cHS4 (chicken hypersensitive site 4) transcriptional insulators. mCherry-hCdt1 labels G1 and early S phase nuclei in red, and mVenus-hGem labels S, G2 and M phase nuclei in green. White indicates co-existing fluorophores. Note loss of fluorescence (black) during cytokinesis. **c** Representative confocal image of a *FUCCI2* mouse in diestrus. 6 positions were imaged over 10 h. Mammary ducts are identifiable by the dense mCherry labelled nuclei. Note the local low fluorescent intensity by light scattering through the overlying mammary fat pad, that forms a black fishnet-like pattern. Scale bar represents 50 µm. Representative of 4 mice. **d** Time-lapse high magnifications of optical sections at the indicated areas in **c** showing two examples of cell cycle transitions from S/G2 to M (upper panel) and from G1 to S phase (lower panel). Insets show a high magnification of the actively cycling cell. SHG is second harmonic generation to visualize collagen I fibers. All scale bars represent 20 µm. **e-f** Characterization of *FUCCI2* fluorescence intensity in the mammary gland over time. **e** Top, optical sections of a duct with an actively cycling cell (inset). Bottom, intensity map of mCherry and mVenus fluorescence from the same duct. Note the high fluorescence background in the ductal lumen and surrounding fatpad in the mVenus channel. All scale bars represent 20 μm. **f** Intensity quantifications of the duct in **e** over the duration of the imaging session (Mean ± SD). Dots represent nuclei. Note constant average intensity in mCherry and mVenus and inherently low mVenus fluorescence intensity

**Fig. 5:**
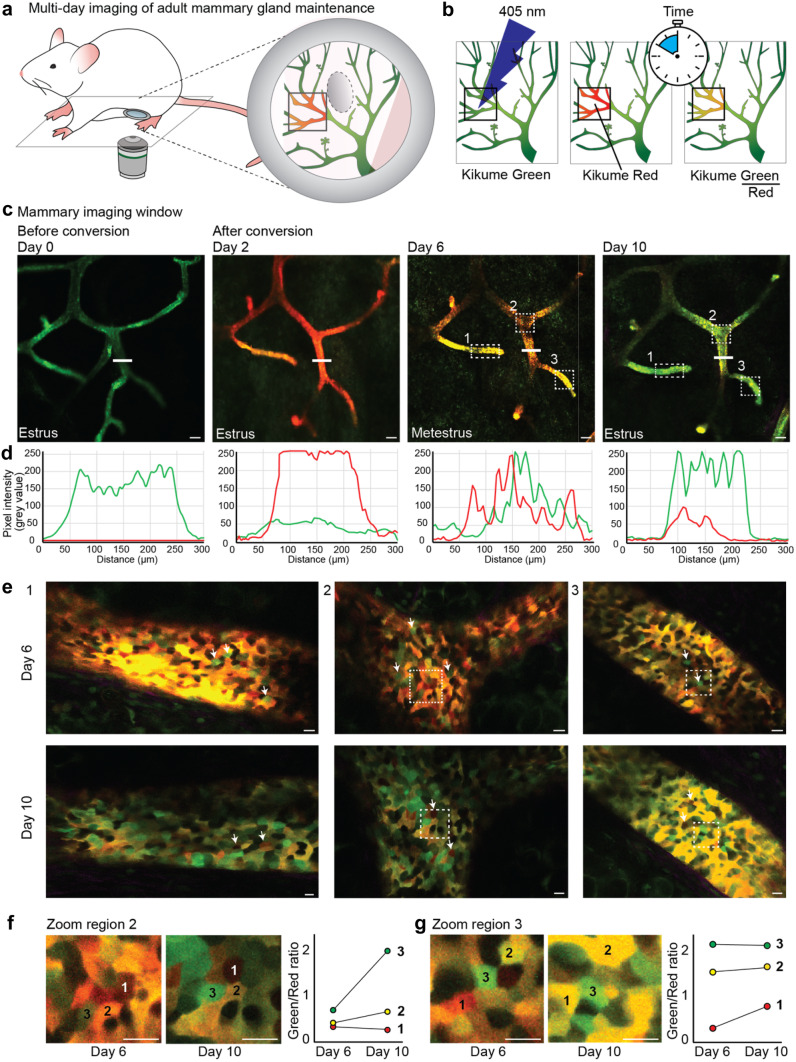
Local proliferative heterogeneity during the estrous cycle in the adult mammary gland using the KikGR mouse model**. a** Cartoon of an adult KikGR mouse, in which a large area of the mammary gland (in green) is photo-converted (red area). **b** Cartoon illustrating the workflow of KikGR photo-conversion of small areas in the mammary ductal tree by exposure to 405 nm laser light. **c** Panels showing confocal images of the same ductal area (maximum intensity projection) in a KikGR mouse over a period of 10 days through a MIW, from left to right 0, 2, 6 and 10 days after exposure to 405 nm laser light. Converted areas show similar dilution of the Kikume red signal, indicating a global and equal turnover rate throughout the epithelium. Estrous stages are indicted in the images. Scale bars represent 100 µm. **d** Line profiles depicting the pixel intensity of green and red signal in the ductal area indicated with the white line in **c**, prior to photo-conversion (left panel), directly after photo-conversion (second panel) and 6 and 10 days after conversion (third and fourth panel). **e** Zoom images (single Z-plane) of different ductal areas indicated with the white boxes in panel **c** 6 and 10 days after photo-conversion, showing wide-spread proliferative heterogeneity at the cellular level. White arrows indicate highly proliferative (green) cells (upper panels) and low proliferative (red) cells (lower panels). Scale bars represent 10 µm. **f** and **g** Zoom image of region 2 **f** and region 3 **g** in which the same cells can be identified 6 and 10 days after photoconversion. Green/red ratio was determined for three neighboring cells, indicated by 1–3, 6 and 10 days after photoconversion (right graphs), illustrating different signal dilution rates of cells within the same tissue neighborhood. Scale bars represent 10 µm. Images and analyses are representative of 4 independent experiments in 4 different mice

First, to visualize the real-time proliferative dynamics, we performed time-lapse imaging using the fluorescent ubiquitination-based cell cycle indicator (*FUCCI2*)-reporter mouse [20] in which cells in G1 phase express mCherry-hCdt-1 and cells during S/G2 phase express mVenus-hGem (Fig. 4a, b, Table 2 and Supplementary Experimental Guide). During the transition from G1 to S phase, cells temporarily express both fluorophores, and during M phase none of the fluorophores are expressed (Fig. 4b). During a 10 h time-lapse we observed both the transition of S/G2 to M phase (Fig. 4c and d, upper panel) and the transition from G1 to S phase (Fig. 4c, d, lower panel, and e), in line with the previously reported long cycling times of most adult ductal cells [10]. However, already at the beginning of the IVM experiment, mVenus-positive cells were drastically underrepresented (Fig. 4c), in contrast to previous ex vivo measurements [5]. Intensity measurements of the *FUCCI2* fluorophores showed weak fluorescent intensity levels of the mVenus fluorophore with considerable background levels in the ductal lumen and adipocytes (Fig. 4e). Throughout imaging, the signal intensity remained stable, and S phase entries were observed also later in the experiment. This indicated that the low number of mVenus-positive cells was due to the *FUCCI*2 detection rather than biological perturbations through the imaging set up (Fig. 4e, f). Thus, in contrast to the skin [21], the mammary gland might be less suitable for intravital *FUCCI2* surveillance. We concluded that IVM in *FUCCI2* mice is limited to ducts closer to the surface, and a subpopulation of mVenus-high cells, whereas deeper tissue layers are optically inaccessible due to the scattering of the fatty tissue surrounding the mammary epithelium and the overall low mVenus fluorescence intensity (Table 2). In an alternative approach for the visualization of single cell dynamics, we analyzed Kikume Green–Red (KikGR)-reporter mice [22, 23] with a MIW (Fig. 5a, Tables 1, 2, and Supplementary Experimental Guide). The KikGR protein is a bright fluorophore that undergoes violet-light induced green-to-red photo-conversion, and was previously used to perform in vivo cell lineage tracking experiments in other organs [22, 23]. After conversion from green-to-red, the green/red ratio can be used as a proxy for the proliferative activity of a cell, in which proliferative cells will dilute the red signal much faster than low proliferative cells (Fig. 5b). It is important to note that the red signal will slowly dilute out due to protein turnover. Because this dilution rate is expected to be similar for all cells, the relative green/red ratio differences can be used to assess proliferative heterogeneity within a tissue over time. We exposed parts of the adult mammary gland to 405 nm laser light and subsequently followed the converted area over a period of 10 days through a MIW (Fig. 5c). Using line intensity profiles we confirmed complete conversion of the KikGR protein, and over time we observed a gradual loss of red signal and recovery of the green signal (Fig. 5d). Interestingly, the overall gradual loss of the red signal in the converted areas was similar throughout all ductal compartments, including branch points, ducts and ductal ends (Fig. 5e and Supplementary Fig. 4a, b). This suggests an equal turnover rate of the different cell populations throughout the mammary gland regardless of their location within the ductal tree, in line with previous ex-vivo analyses of *FUCCI2* mammary glands [5, 10]. These dynamics stand in sharp contrast to the pubertal situation, where the proliferation is solely focused within the terminal end buds.

Next, we assessed the turnover rate of the individual cells within the different ductal structures. The majority of the cells showed a similar dilution rate of the red signal, indicating a similar proliferation rate (Fig. 5e and Supplementary Fig. 4c). Strikingly, some cells showed immediate dilution of the red signal 6 days after conversion, indicating high proliferative activity (Fig. 5e, upper panels, arrows, Supplementary Fig. 4b, c), whereas other cells retained their red signal over the entire period of 10 days, indicating a low proliferative state (Fig. 5e, lower panels, arrows, Supplementary Fig. 4b, c). Qualitative assessment indicated that high proliferative and low proliferative cells were equally distributed over the converted areas suggesting a uniform but heterogeneous turnover rate within the ductal epithelium during the estrous cycle (Fig. 5c, d). However, at the local level, neighboring cells showed remarkable differences in their green/red ratio 6 and 10 days after photo-conversion, indicating proliferative heterogeneity (Fig. 5e–g). To further characterize this striking local proliferative heterogeneity, we quantified the change of green/red ratio within the same three neighboring cells 6 and 10 days after photo-conversion (Fig. 5f, g, right graphs). The green/red ratio dynamics over time, as a proxy for proliferative activity, were indeed highly variable between neighboring cells within the same micro-environment (Fig. 5f, g). To confirm these findings, and to test for potential perturbations of tissue homeostasis by the IVM procedure, we performed ex vivo analyses of 10 h 5-ethynyl-2-deoxyuridine (EdU) pulse-chases during the different stages of the estrous cycle (Supplementary Fig. 5a). In support of the in vivo data, we found widespread proliferation throughout the ductal tree (Supplementary Fig. 5a, b). Together these results indicate that the mammary gland shows an overall uniform, but locally heterogeneous proliferative activity within the mammary gland during the estrous cycle.

## Adult Mammary Gland Tissue Architecture is Extremely Stable in the Long-term

**Fig. 6:**
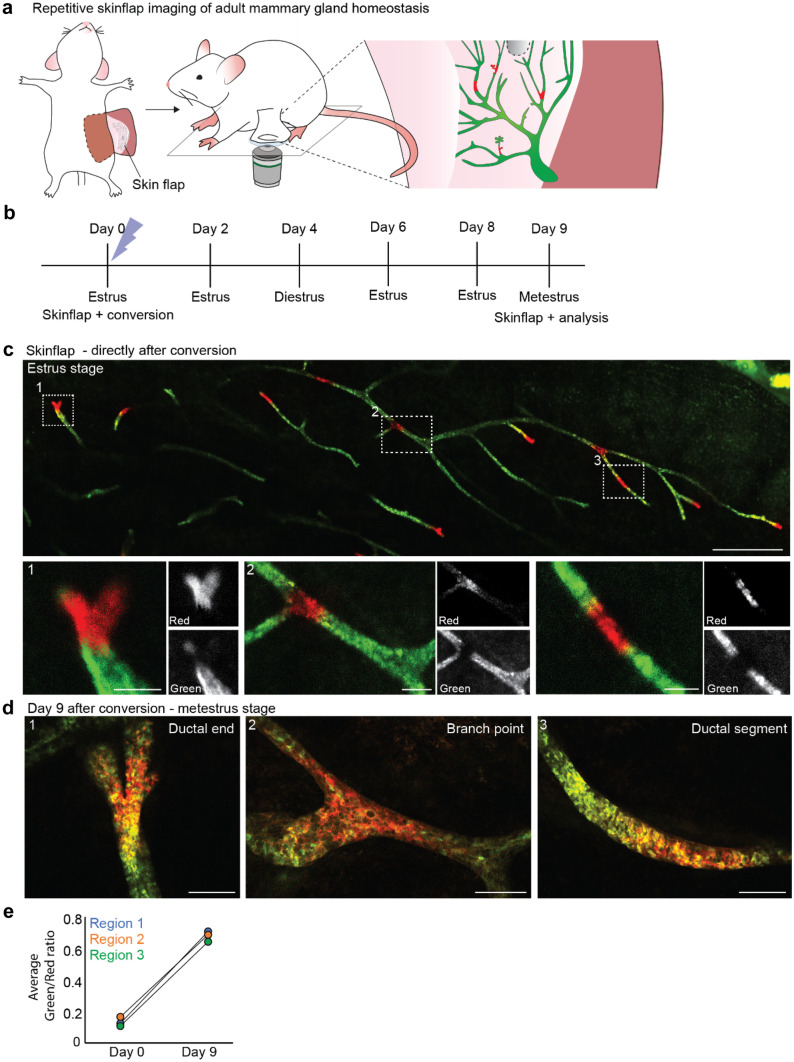
Repeated skin flap during the estrous cycle demonstrates limited cellular rearrangements and ductal stability. **a** Cartoon of an adult KikGR mouse in which small areas of the mammary gland were photo-converted using the skin flap method. **b** Timeline of the repeated skin flap experiment over the course of an entire estrous cycle. **c** Confocal overview image (single Z-plane) of the visible area of the mammary gland using a skin flap (upper panel) and zoom images of the indicated converted areas (lower panels). Mammary gland is shown directly after the photo-conversion of small areas. Scale bars represent 1 mm (overview image) and 100 µm (zoom images). **d** Zoom images of the regions indicated with white boxes in panel **c** 9 days after photo-conversion (single Z-planes). Converted areas stayed cohesive over time, and no difference in dilution rate is observed between different regions (ductal end, branch point, and ductal segment respectively) indicating similar turnover rates throughout the gland. Scale bars represent 100 µm. **e** Average green/red ratio of the three photo-converted regions depicted in **c** and **d** directly after photo-conversion (Day 0) and after a period of 9 days, demonstrating an equal turnover rate throughout the different ductal compartments. Images are representative of 4 independent experiments in 4 different mice

Next, we addressed how the continuous cycling and local remodeling of the mammary gland affects the overall tissue architecture of the mammary gland. To assess the long-term stability of the mammary gland, we established a repeated skin flap technique using the KikGR reporter mouse model (Supplementary Experimental Guide and Table 1, 2). First, we performed a skin flap imaging session during which we imaged a large area of the mammary gland (Fig. 6a). Subsequently, we locally converted small sections of the mammary gland by exposure to 405 nm laser light at estrus stage and closed the skin flap after the imaging session (Fig. 6b, c, and Supplementary Fig. 6a). To check for local stability of the mammary gland we re-opened the skin flap one full estrous cycle after the first imaging session and traced back the same converted areas (Fig. 6b, d and Supplementary Fig. 6b). In line with the previous experiments all converted areas showed similar dilution of the red signal, indicating a uniform homeostatic turnover within the mammary gland, whereas at the cellular level large proliferative heterogeneity could be observed (Fig. 6d, e, and Supplementary Fig. 6b). Moreover, the local converted regions stayed cohesive and similar in size compared to Day 1, demonstrating that the local tissue architecture is stable and large-scale cellular mixing does not occur over one estrous cycle (Fig. 6d and Supplementary Fig. 6b).

**Fig. 7:**
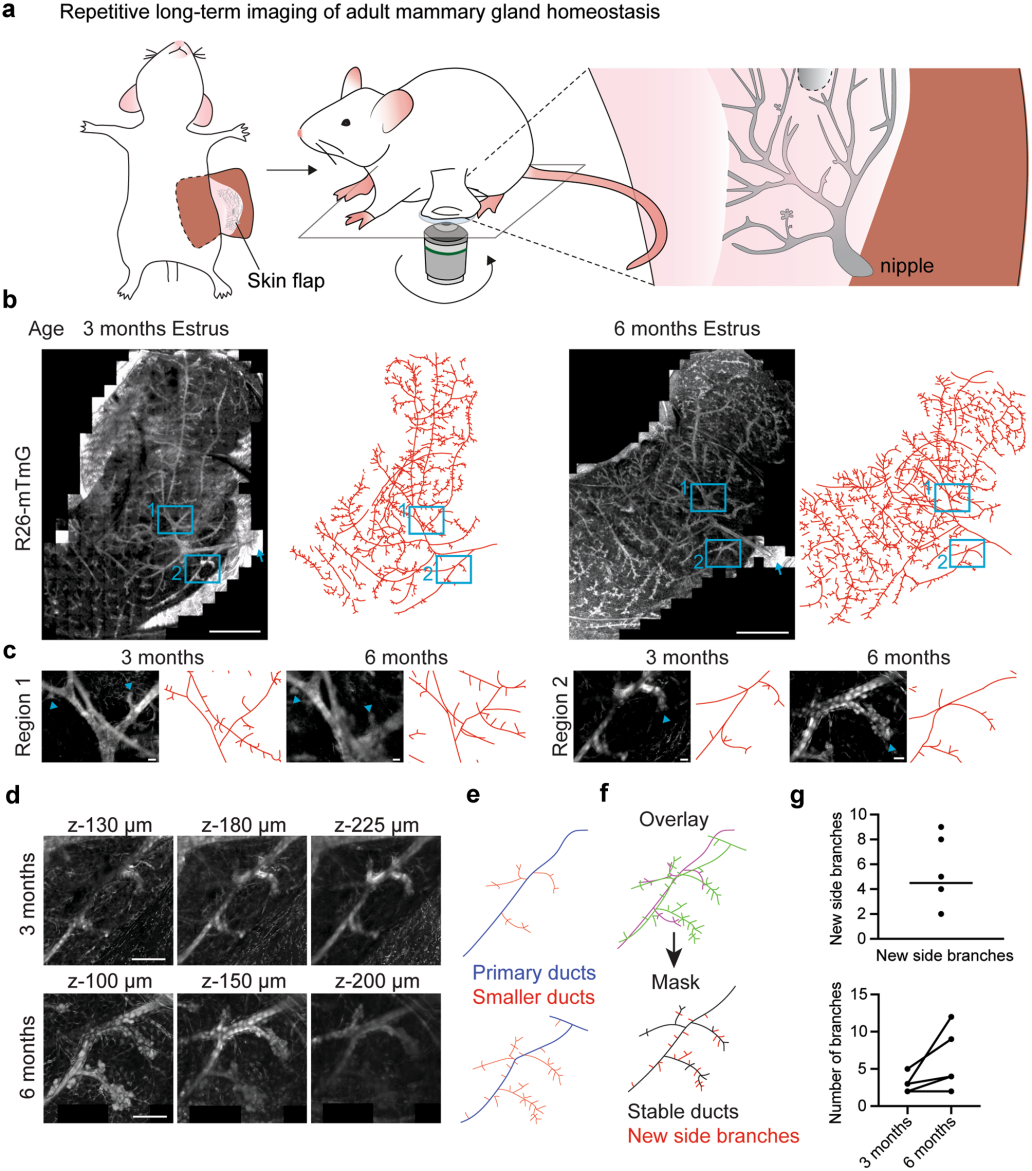
Intravital microscopy of long-term adult mammary gland turnover reveals architectural stability. **a** Cartoon of the repetitive skin flap technique. The nipple is used as a landmark to find the same region in the subsequent imaging session. **b** Left, 4th mammary gland of a 12-week old *R26-mTmG* mouse in estrus and reconstructed ductal map. Scale bar represents 2.5 mm. Right, same mammary gland 3 months later in estrus. Scale bar represents 3 mm. Arrows demarcate nipple. Representative of 4 mice. **c** High magnifications of the indicated areas in **b** showing regions with unchanged tertiary branches (region 1) or local branch expansion (region 2). Arrowheads demarcate tertiary branches. All scale bars represent 100 µm. **d** Zoomed optical sections of different z-levels at region 2. All scale bars represent 500 μm. **e** Maps of ductal branching derived from the images in **d**. Primary ducts (blue) are directly linked to the main duct. Side branches are coloured red. **f** Top, overlay of the branch maps in **e**. Mauve, 3 months; green, 6 months. Bottom, consensus map of stable ducts (black) that are present in both timepoints and newly formed branches (red) that were only found at 6 months. **g** Quantification of new side branches per duct after 6 months (top) and comparison of branches per duct between 3 and 6 months (below)

It is unclear how the repeated cycles of ductal budding and regression in each estrous cycle affect the geometry of the ductal tree over longer time periods. To surveil ductal organization during adult homeostasis, we extended our repeated skin flap IVM technique over 3 months, and imaged the mammary tree starting at estrus stage from the nipple, which was used as reference landmark in subsequent analyses (Fig. 7a). We used *R26-mTmG* mice, which due to their high ubiquitous membranous fluorescence enabled us to visualize ductal architecture into deeper tissue layers, and to map large areas of the ductal tree (Fig. 7b, Table 2). Ducts in deeper tissue layers were not optically accessible in each imaging session and care needs be taken in the reconstruction of ductal organization. Our analyses suggested that the organization of higher order ducts remains largely unaffected by the short-term remodeling of the estrous cycle (Fig. 7b). Most tertiary branches were still present after three months, but had undergone variable changes in size and morphology (Fig. 7c). Interestingly, some ductal segments had gained new tertiary branches, suggesting a regional heterogeneity in the extent of estrous budding (Fig. 7d–g).

## Discussion

Here we established a toolbox for the quantitative analysis of cellular dynamics in the pubertal and adult mammary gland. We explored three experimental avenues that provide optical access to the mammary gland with time scales ranging from consecutive imaging over several hours/days to repeated imaging of the same area over months. Implantation of an MIW allows to revisit the same areas over several days whilst keeping the mammary gland in an unperturbed environment. The replaceable lid can further improve imaging quality by ensuring constant high signal resolution and light penetrance over time. Both MIW approaches are limited in their imaging field by the dimensions of the window coverglass insert (Table 1). The skin flap technique is an attractive option when larger areas need to be analyzed, for example to capture rare events or the overall architecture of the mammary tree. The skin flaps can be repeated over time to allow revisiting of the same mammary gland. However sufficient time needs to be allocated in between surgeries to allow recovery of the skin (Table 1).

Our technical analyses argue that the experimental goal determines the imaging avenue to be used, but also the mouse model to be chosen. All IVM approaches tested here provide sufficient resolution to visualize the behavior of single cells in the mammary epithelium. The constitutive strong membranous fluorescence in the *R26-mTmG* mouse is an ideal choice to follow single cells, but is also strong enough to capture entire ducts in deeper tissue layers, necessary for mapping the mammary tree. If single cells are to be followed over intermittent imaging sessions, the local recombination of a subset of cells from red to green membrane fluorescence, for example stochastically with a *R26-CreERT2* allele, can be useful (Table 2). However, when the aim is to follow specific ductal subpopulations, for example basal versus luminal cells, over time, it is advisable to use (CreERT2-) reporter lines specifically labelling the population of interest.

The quantitative output of our IVM approaches is multifold in that it allows the quantification of proliferation, movements and rearrangements from single cell level to tissue scale. For example, constitutive imaging of single recombined green cells in the unrecombined red tissue context of the *R26-mTmG* mammary gland, allows to track movements and cell divisions over time. The *FUCCI*2 and KikGR mouse models offer more targeted insights into the dynamics of neighboring duct cells, but come with their own shortcomings (Table 2). The *FUCCI*2 cassette, whilst giving excellent performance in the intravital surveillance of the murine skin [21], displays an overall low fluorescence intensity in the mammary gland, where even ducts close to the tissue surface are overshadowed by overlying adipocytes that cast a ‘fishnet-like’ pattern on the duct. Furthermore, the low mVenus intensity and high green autofluorescence levels from the mammary fat pad and ductal lumen compromise the detection of S/G2 cells. Thus, in comparison with previous ex vivo analyses [5], we could only identify a subpopulation of mVenus-high cells. The optical properties of the KikGR model are more suitable for the mammary gland. However, the protein is mosaically expressed and more in-depth characterization is necessary to identify the KikGR-labelled population. In the KikGR model, cycling cells, as indicated by the dilution of photoconverted protein, are more abundant than in the *FUCCI*2 mouse. This conclusion, however, depends on the assumption that protein turnover is more affected by cell division than by heterogeneous synthesis and degradation of KikGR protein, and we recommend to corroborate the IVM data with direct analyses of proliferative activity, for example by EdU pulse chase experiments. In conclusion, we advise to carefully evaluate each fluorescent mouse model for its performance in the mammary gland, before commencing with IVM experiments. Specific points to ensure are robust fluorophore expression in the target cell population, a high enough fluorescence intensity and signal-to-noise ratio, and a constant signal intensity over time.

The efforts to establish the experimental setup are repaid by the achievable data quality. IVM allows the unique possibility to capture and quantify cell biology in vivo in the native tissue context. The stochastic tracing employed in our study can be taken further to the stochastic or lineage-targeted perturbation of signaling pathways, which poses an attractive solution to disentangle the molecular mechanisms at play during mammary gland development, homeostasis and remodeling. Therein, ‘treated’ and ‘control’ cell populations can be compared side-by-side in the same mammary gland, reducing biases and errors due to differential sample handling. Finally, the images obtained through a time-lapse or multi-day experiment provide 4-dimensional (xyzt) data at a cellular level, which can greatly reduce the required number of experimental animals. However, we do recommend to repeat experiments on sufficient replicates. To ensure reproducibility between individual animals, we advise to repeat experiments in 3 to 5 animals as a starting point, given that the animals are imaged throughout the same stages of the estrous cycle.

Previous work has pioneered both the ex vivo [24] and in vivo surveillance of many stages of mammary gland physiology, including pregnancy [25–27] and breast cancer [21, 22, 29, 30], demonstrating the excellent suitability of the mammary gland to IVM. In this study, we focused on the pubertal and adult homeostatic mammary gland. Pubertal mammary gland development is characterized by constant proliferation that drives ductal elongation and branching. Previous static time course analyses showed that this branching morphogenesis is orchestrated from the TEB compartment [2, 6]. Using a MIW, we rendered the TEB niche optically accessible for continuous surveillance by confocal microscopy. By comparative time-lapse imaging of cell behavior in the TEB and adjacent intermediate ductal segments, we showed that high cell proliferation, as well as continuous and random cellular rearrangements fuel TEB activity and branching. A recent modelling approach suggested that these random rearrangements, partly driven by cellular proliferation, may provide an important mechanism for MaSCs to regain a favorable position within the stem cell niche [31]. Previous studies based on ex vivo time-lapse imaging and modelling also reported cellular flux from the basal to luminal region by cap cells, providing yet another source for cellular mixing within the TEB [32, 33]. At the molecular level, the TEB cells were shown to be a highly heterogeneous pool of cells. Previous single cell mRNA sequencing analysis of micro-dissected TEBs revealed that the TEB cells are highly heterogeneous in their expression profile [2]. Interestingly, this heterogeneity and cellular mixing was also predicted in several theoretical models of the TEB, in which it was shown that different cellular phenotypes with different proliferative capacities were randomly distributed within the TEBs [34, 35]. Future studies will be required to elucidate the mechanisms that drive the continuous cell mixing, the remarkable phenotypic cellular heterogeneity within the TEBs, and their biological relevance during branching morphogenesis.

The pubertal TEB niche exhausts its proliferative potential by the onset of adulthood and is replaced by blunt ductal endings, indicating that the pubertal MaSCs in the TEBs only serve as a transient stem cell pool during ductal morphogenesis. As a consequence, the TEBs cannot be the location of long-term progenitors or MaSCs during adult life. Several studies have speculated on the location of MaSCs in the adult mammary gland. For example, it has been suggested that adult MaSCs are located in the suprabasal layer (between the basal and luminal cell layer), near the branch points [36, 37], or the proximal area of the gland [38]. In contrast, by long-term administration of synthetic nucleosides it was shown that proliferation is scattered throughout the ductal epithelium [10] suggesting equal distribution of MaSCs driving long-term turnover in the adult mammary gland. Because the exact location and niche of the adult MaSCs remains unexplored, it is unclear if all ductal segments share a similar proliferative activity, or if the ductal tree comprises quiescent and proliferative zones. In addition, the cellular dynamics of alveolar bud formation during the estrous cycle are little understood due to the technical limitation to static analyses. Here, using time-lapse imaging of *FUCCI2* cell cycle reporter mice, we could identify cycling cells throughout ductal segments. To assess if there are regional proliferative hotspots, we combined the KikGR cell division recorder with repeated imaging of the same ductal regions. We tracked proliferation in intermediate segments, branch points and tertiary branches over one complete estrous cycle. KikGR ratio dilution identified highly active (green) and quiescent cells (red) in all regions, and at a similar frequency, suggesting an overall similar proliferative activity throughout the ductal tree. KikGR converted areas largely maintained their size and shape, which indicates limited cell mixing within the ducts during adulthood. Together, these results suggest that the different cell populations are equally distributed over the ductal tree. At the cellular level however, we observed extreme differences in the KikGR ratio dilution, where neighboring cells showed opposite proliferative behavior ranging from highly active (green) to quiescent (red), suggesting a locally heterogeneous proliferation capacity within the same neighborhoods. Indeed, previously it was shown that the different cell types within the mammary gland respond differently to the hormonal cues during the estrous cycle [5, 10]. Moreover, experiments using label retention methods already hinted towards regional proliferative differences within the adult mammary gland, but at the same time reported equal distribution of label-retaining cells over the entire ductal tree [39]. Together these data indicate that whilst at the cellular level neighboring cells are highly heterogeneous, adult ducts share an overall homogeneous proliferative activity.

To assess the implications of this proliferative heterogeneity and local turnover dynamics for ductal architecture, we followed ductal organization in the same adult mammary glands over 3 months. Despite the continuous and active cycling of cells throughout the ductal tree, large ductal segments were highly stable in the long term. The overall organization of higher order ducts did not change, however morphological alterations were observed in the tertiary branches, in line with their remodeling throughout the estrous cycle [40]. The extent of these changes varied among areas, and contributed to regional increases in ductal complexity after three months.

Further studies are needed to draw detailed maps of the dynamic spatiotemporal activity of cycling cells in the adult mammary duct. Especially duct cell progenitor status, cell fate, and the regulation by the estrous cycle need to be examined in the unperturbed tissue environment. A range of state-of-the-art fluorescent reporter mouse models has been established in the previous years to visualize live cell behavior and fate changes. We find that both the pubertal and adult mammary glands are well amenable to the light microscopic analysis of single cell behavior. Intravital imaging techniques, specialized for short-term continuous imaging or long-term surveillance, offer unprecedented insight into mammary gland biology in vivo*,* and will contribute to shed light on the mechanisms underlying ductal homeostasis and remodeling.

## Materials and Methods

### Mice

All mice were females, housed under standard laboratory conditions, and received food and water ad libitum. All experiments were performed in accordance with the guidelines of the Animal Welfare Committee of the Royal Netherlands Academy of Arts and Sciences, The Netherlands and the Netherlands Cancer Institute (NKI), Amsterdam, The Netherlands. To image pubertal mammary gland development, *R26-CreERT2*;*R26-mTmG* mice of mixed background were injected intraperitoneally with 0.2 mg tamoxifen (Sigma Aldrich), diluted in sunflower oil to activate Cre recombinase at 3 weeks of age. To visualize adult mammary gland dynamics, unrecombined *R26-mTmG* (C57BL/6) mice, fluorescent ubiquitination-based cell cycle indicator (*FUCCI2,* mixed background) and Kikume Green–Red (*CAG-KikGR,* C57BL/6 background) mice were used older than 10 weeks of age. To ensure continuous cycling, adult female mice were regularly exposed to male pheromones either by opening the cages next to each other, or by placing some male bedding in the cage with female mice. Experiments were not randomized, sample size was not determined a priori, and investigators were not blinded to experimental conditions except where indicated. For more details on the mouse models and their use for IVM, see Table 2.

### Window and Skin Flap Surgeries

All surgeries were carried out under anesthesia with 1.5%-2% isoflurane/air. Duratears were used to prevent dehydration of the eyes. Surgical sites were shaved and cleaned with betadine. Window implantation was carried out as described [18]. In brief, a 10 mm skin incision was placed above the 4^th^ mammary gland and the skin loosened from underlying tissue by blunt dissection. Non-absorbable suture was placed in loops around the incision, the sterilized window inserted and sutures tightened and tied. For repeated imaging with a skin flap, a midline incision was placed at height of the 4^th^ mammary gland and two lateral incisions anterior and posterior of the 4^th^ mammary gland. Care was taken not to disturb the mammary fat pad. The skin was loosened from the underlying mammary gland with blunt dissection and flipped, and the mouse turned so that the exposed mammary gland could be imaged with an inverted microscope. The mouse was placed on a custom-designed imaging plate with an inserted 24 mm × 50 mm coverglass. The 4^th^ mammary gland was placed on the glass and covered with sterile gauze soaked in PBS. Parafilm was taped over the gland to stabilize its position. After imaging, the skin was closed with non-absorbable suture. For analgesia mice received carprofen in drinking water (1 day before surgery to 72 h after) and subcutaneous injection of buprenorphine. For detailed information on the surgical procedures and their use for IVM, see Supplementary Experimental Guide and Table 1.

### Intravital Imaging of Mammary Glands

Mice were anesthetized using isoflurane inhalation (1.5%–2% isoflurane/air mixture) and placed in a facemask within a custom designed imaging box. Isoflurane was introduced through the facemask and ventilated by an outlet on the other side of the box. The imaging box and microscope were kept at 34 °C by a climate chamber surrounding the entire stage of the microscope including the objectives. Imaging was performed on an inverted Leica SP8 Dive system (Leica Microsystems, Mannheim, Germany) equipped with 4 tunable hybrid detectors, a MaiTai eHP DeepSee laser (Spectra-Physics) and an InSight X3 laser (Spectra-Physics) using the Leica Application Suite X (LAS X) software. All images were collected at 12 bit and acquired with a 25 × water immersion objective with a free working distance of 2.40 mm (HC FLUOTAR L 25x/0.95 W VISIR 0.17).

To image pubertal development *R26-CreERT2;R26-mTmG* mice were intraperitoneally injected with 0.2 mg tamoxifen per 25 g body weight diluted in sunflower oil (Sigma) at 3 weeks of age. At 5 weeks of age, a skin flap was performed (for time-lapse imaging) or a mammary window was inserted near the fourth and fifth mammary glands (for details, see [18] and Supplementary Experimental Guide) for multiday imaging. To obtain time-lapse movies, TEBs and ducts were imaged every 20–30 min using a *Z*-step size of 3 μm over a minimum period of 8 h. For multiday imaging, the same imaging fields were retraced during subsequent imaging sessions using the imaging coordinates and collagen I structure of the first imaging session. GFP and Tomato were excited with a wavelength of 960 nm and detected between 500–550 nm (GFP) and 560–650 nm (Tomato).

To detect proliferating cells in the adult mammary gland, the 4^th^ mammary gland of *FUCCI2* mice was exposed with a skin flap and imaged continuously over 10 h. Mice received nutrition by subcutaneous injection of 100 ul/h Nutriflex® (Braun). The *FUCCI2* fluorophores were excited simultaneously at 960 nm and detected between 490–560 nm (mVenus) and 600–690 nm (mCherry). SHG was used to detect collagen I. Ducts were imaged every 30 min in bidirectional mode, at 600 Hz, with a local z-scan with 2 μm z-step size.

For multi-day imaging of the adult mammary gland using the *CAG::KikGR* mice, a MIW was implanted on one 4th mammary gland or a skin flap surgery was performed. Next, an overview scan of the full visible tissue area was acquired. After this, areas of interest were defined by drawing a region of interest (ROI). For each defined ROI, a Z-stack was made with step sizes of 1 μm. Photo-conversion was performed using the 405 nm laser line at 2% power (equivalent to 115 μW/mm2), 600 Hz, 25 × magnification, and 1024 × 1024 pixels with a pixel dwell time of 600 ns. Detailed three-dimensional Z-stacks were made of the areas containing the converted tissue areas with a Z-step size of 0.5–1 µm. Both Kikume Green and Kikume Red were excited with 960 nm, and collected between 490–550 nm (KGreen) and 560 – 700 nm (KRed). For the multi-day imaging through the MIW, tile scans were performed every other day. For the skin flap imaging, the skin flap was closed with sutures after the imaging session and after one full estrous cycle the skin flap was re-opened for the second imaging session. The same imaging fields were retraced during subsequent imaging sessions using the imaging coordinates and collagen I structure of the first imaging session.

For mapping of the mammary ductal trees, the 4th mammary gland of adult *R26-mTmG* mice was imaged repeatedly with a skin flap. tdTomato was excited at 1040 nm and detected at 540 nm – 790 nm. All visible ducts were imaged together in one tile-z-scan with 0.75 × zoom, a z-step size of 10—20 μm, bidirectional mode, 600 Hz scan speed, and a pixel size of 2 μm. These parameters allowed to scan large regions of up to 2 cm^2^ in less than 3 h.

### Estrous Cycle Staging

To determine the estrous cycle stage of the mice, a vaginal swab was collected prior to each intravital imaging session (method described in [41]). In short, the vagina was flushed using a plastic pipette filled with 50 µl PBS, and transferred to a dry glass slide. After air drying, the slide was stained with Crystal Violet and the cell cytology was examined using a light microscope.

### EdU Pulse-chase and Whole Mount Analysis

Estrous stage of adult female mice was detected and n = 2 mice per stage (proestrus, estrus, and metestrus/diestrus) were injected intraperitoneally with 0.5 mg 5-ethynyl-2-deoxyuridine (EdU, Invitrogen) diluted in PBS. Mice were euthanized 10 h after EdU injection and the third, fourth and fifth mammary glands were collected and processed as whole-mount glands. First, mammary glands incubated in a mixture of collagenase I (1 mg/ml, Roche Diagnostics) and hyaluronidase (50 μg/ml, Sigma Aldrich) at 37 °C, fixed in periodate–lysine–paraformaldehyde (PLP) buffer (1% paraformaldehyde (PFA; Electron Microscopy Science), 0.01 M sodium periodate, 0.075 M L-lysine and 0.0375 M P-buffer (0.081 M Na2HPO4 and 0.019 M NaH2PO4; pH 7.4) for 2 h at room temperature. 5-ethynyl-2-deoxyuridine (EdU) cell-proliferation staining of whole-mount mammary glands, a click-it stain (Click-iT EdU Alexa647, Invitrogen) was performed according to the manufacturer’s instructions. Nuclei were stained with DAPI (0.1 μg/ml; Sigma-Aldrich) in PBS. Glands were washed with PBS and mounted on a microscopy slide with Vectashield hard set (H-1400, Vector Laboratories). Whole-mount mammary glands were imaged on an inverted Leica SP8 confocal microscope. DAPI was excited at 405 nm, and collected at 440-470 nm, Alexa647 was excited at 633 nm, and collected at 650–700 nm. The whole-mount mammary glands were imaged in 3D using a large scale tilescan with a total Z-stack of around 200 µm with a Z-step size of 5 µm. For analysis, 3D tile-scan images of the whole-mount glands were taken and the number of EdU^+^ ducts was scored.

### Image Analysis

Three-dimensional overview tile scans were stitched and processed in the real-time Rendering LAS X 3D Visualization module (Leica Microsystems, Mannheim, Germany). Time-lapse three-dimensional volumes were corrected for XYZ-shift using the Huygens Object Stabilizer module (Scientific Volume Imaging). Individual cells were tracked using the MTrack2 plugin in ImageJ (Stuurman, N., Schindelin, J., Elliot, E., and Hiner, M., https://imagej.net/MTrack2/). Nuclear segmentation and intensity measurements were performed with Imaris 9.6 (Bitplane) using the surface reconstruction module. Ductal trees were reconstructed manually by outlining the ducts.

## Supplementary Information

Below is the link to the electronic supplementary material.

Supplementary file1 (DOCX 372 KB)

Supplementary file2 (DOCX 7771 KB)

## Data Availability

All data and material are available upon reasonable request from colinda.scheele@kuleuven.be or j.v.rheenen@nki.nl.
