# Supplementary material for: An Intravital Microscopy Toolbox to Study Mammary Gland Dynamics from Cellular Level to Organ Scale
Source: J Mammary Gland Biol Neoplasia. 2021 May 4;26(1):9–27. doi: 10.1007/s10911-021-09487-2 (PMC8217050; doi:10.1007/s10911-021-09487-2)
Supplement: Supplementary file 1 — Supplementary file1 (DOCX 372 KB) [file 10911_2021_9487_MOESM1_ESM.docx]

**In this supplementary guide we provide step-by-step instructions for the implantation of imaging windows, the skin flap technique and repeated intravital microscopy (IVM). More information on the windows and IVM setup can be found in** [**https://www.nature.com/articles/nprot.2013.026**](https://www.nature.com/articles/nprot.2013.026) **and** [**https://www.tandfonline.com/doi/full/10.4161/intv.29917**](https://www.tandfonline.com/doi/full/10.4161/intv.29917) **All animal procedures should be carried out in adherence to national and institutional legislation.**

1. **Implantation of the mammary imaging window**
2. **Repeated intravital microscopy with the imaging window**
3. **Imaging the mammary gland via the skin flap procedure**
4. **Mouse nutrition during IVM**

**1) Implantation of the mammary imaging window**

**HEALTH AND SAFETY**

Beware of needlestick injuries, scratch and bite wounds when handling the mouse. Take caution when working with 80% ethanol and VirkonS; avoid contact with skin and eyes, and avoid inhalation.

**REQUIRED MATERIALS**

- VirkonS 1%
- Analgesia as per institutional guidelines
- Rimadyl
- Duratears
- Isoflurane + anesthesia induction box
- 1 ml syringes with needle
- Sterile drape
- Heating mat
- Gloves
- Razor blades
- Germinator
- Sterile surgery tools in box (straight scissors, blunt forceps, sharp forceps, bended forceps, westcott type scissors, needle holder)
- Sterile PBS
- Ethanol 80%
- Sterile ethanol prep pads
- Betadine solution, sterile betadine prep pads
- Super glue (Bison)
- Toothpicks
- Mammary imaging window (MIW) or MIW with replaceable lid (R.MIW)
- 10 or 12 mm glass coverslip
- Vaseline
- Sterile gauze
- Sutures
- Sterile cotton swabs

**PREPARATION**

1. Put on gloves
2. Turn on the biohazard cabinet and clean it. Put sterile drape on the working area.
3. Put all tools in the biohazard cabinet. Wipe everything with VirkonS before introducing them in the biohazard cabinet.
4. Turn on the heating mat and Germinator.
5. Sterilize the instruments using the Germinator and lay them out.
6. Prepare the injection fluids.
7. Prepare the mammary imaging window by gluing a glass coverslip on top:
   1. MIW:
      1. Place the MIW with the window groove facing upwards.
      2. Using a toothpick, put small dots of glue all the way around the groove.
      3. Carefully place a 12mm glass coverslip on top of the insert.
      4. Use a toothpick to position it and then push down.
      5. After 10 - 30 min of air drying, disinfect the window in 80% Ethanol for 30' - 1 hr (not longer or the glue may dissolve.
      6. Remove extra glue either by briefly soaking the insert in acetone or by using a cotton tip soaked with acetone to wipe the window.
   2. R.MIW:
      1. Place the R.MIW lid so that the arms on the outside of the ring are sloping downwards.
      2. Using a toothpick, put small dots of glue all the way around the rim.
      3. Carefully place a 10 mm glass coverslip on top of the insert.
      4. Use a toothpick to position it and then push down - make sure none of the holes in the arms are covered up.
      5. After 10 - 30 min of air drying, disinfect the window in 80% Ethanol for 30' - 1 hr (not longer or the glue may dissolve.
      6. Remove extra glue either by briefly soaking the insert in acetone or by using a cotton tip soaked with acetone to wipe the window.
8. Administer preoperative analgesia.

**SURGERY**

1. Anesthetize the animal using isoflurane.
2. Cover the eyes with Duratears.
3. On the animal preparation side (not on top of the sterile drape in the cabinet), shave the area above and around the 4^th^ mammary gland using a razor blade. Remove hairs.
4. Move the mouse to the (sterile) surgical area within the biohazard cabinet, place it on its back.
5. Change gloves, then wet your gloved hands with VirkonS, wipe off using a tissue, spray with 80% ethanol and let air-dry.
6. Cover the mouse with a sterile cover sheet or sterile tissue with a hole cut in it, leaving the surgical area visible.
7. Disinfect the skin with betadine and 80% ethanol.
8. Make a clean cut on top of the 4th mammary gland without damaging the mammary fat pad or peritoneum.
9. Remove the skin from underlying layers by blunt dissection.
10. Test whether the incision is big enough by inserting the window. Remove window to place the sutures.
11. Place a purse string suture by making loops (approx 5-6), start at one of the corners of the incision (the weakest point).
12. Fit the window inside the incision and pull the loops tight one by one, starting with the farthest loop. Take care that the skin and sutures remain stuck in the MIW groove all around the window.
13. Pull tightly on the ends of the sutures and tie off with surgical knots.
14. For the R.MIW: Apply vaseline to the metal ring of the lid, avoiding the glass coverslip, and then place into the window and tighten the sutures (for image of the R.MIW see images below).
15. Let the mouse recover in a cage half on the heating mat until it is awake.


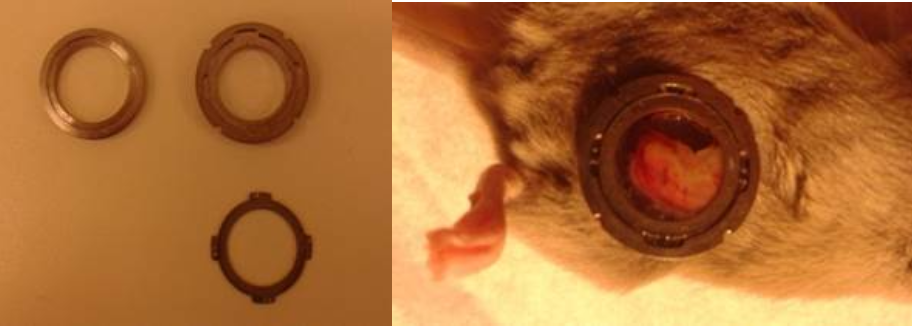
Mammary imaging window (MIW) (l) and mammary imaging window with replaceable lid (R.MIW) (l), placed on the mouse (r).

**REMARKS**

- Follow aseptic practice during surgeries.
- Review the analgesia strategy with your local veterinarian and follow the institutional guidelines.
- Every time before touching a mouse, disinfect hands with VirkonS, wipe off using a tissue, disinfect with ethanol 80% and let evaporate, so that the mouse is as minimally as possible exposed to VirkonS.
- By using a tissue (or other surgery cover) to cover the mouse the surgical area stays cleaner, especially during suturing, when the thread often goes through the mouse' hair.
- Preferably work in couples.
- Leave the anaesthetized mouse on the heating pad as much as possible.
- All surgical instruments are sterile and sterilized between animals using a glass bead sterilizer (Germinator).

**2) Repeated intravital microscopy with the imaging window**

**HEALTH AND SAFETY**

Beware of needlestick injuries, scratch and bite wounds when handling the mouse. Take caution when working with 80% ethanol and VirkonS; avoid contact with skin and eyes, and avoid inhalation.

**REQUIRED MATERIALS**

- VirkonS 1%
- Duratears
- Isoflurane + anesthesia induction box
- Gloves
- Sterile PBS
- Heating mat
- Ethanol 80%
- Sterile ethanol prep pads
- Sterile gauze
- Sterile cotton swabs
- Microscope with climate chamber
- Imaging box
- Nutriflex® special 70/240 (Braun)
- Infuse system
- Infuse needle (flexible needle)
- Tubing
- Parafilm

**PROCEDURE**

1. Sterilize gloves with 80% Ethanol.
2. Anesthetize mouse in the cage with 2% isoflurane (This is reduced after induction to 0.8-1.5% during imaging).
3. Transfer mouse from the induction box to the imaging box, on top of a custom metal inlay with a hole the diameter of the MIW.
4. Put mouse with the head inside the anaesthesia nozzle and position the window on the inlay in such a way that the window falls in the hole.
5. Secure the mouse with parafilm and tape.
6. Place the imaging box in the microscope.
7. Provide proper nutrition during imaging (see guide 4, Mouse nutrition during IVM).
8. Set up the imaging, and keep monitoring the mouse by checking the breathing every 15 minutes, and adjust the % of isoflurane if needed.
9. After completion of IVM, let the mouse recover in a cage half on the heating mat for ~1 hour.
10. Switch of the anaesthesia machine, and clean the imaging box and inlay with VirkonS and ethanol 80%.

**3) Imaging the mammary gland via the skin flap procedure**

**HEALTH AND SAFETY**

Beware of needlestick injuries, scratch and bite wounds when handling the mouse. Take caution when working with 80% ethanol and VirkonS; avoid contact with skin and eyes, and avoid inhalation.

**REQUIRED MATERIALS**

- VirkonS 1%
- Buprenorphine (Temgesic)
- Rimadyl
- Duratears
- Isoflurane + anesthesia induction box
- 1 ml syringes with needle
- Sterile drape
- Heating mat
- Gloves
- Razor blades
- Germinator
- Sterile surgery tools in box (straight scissors, blunt forceps, sharp forceps, bended forceps, westcott type scissors, needle holder)
- Sterile PBS
- Ethanol 80%
- Sterile ethanol prep pads
- Betadine solution, sterile betadine prep pads
- Sterile gauze
- Sutures
- Sterile cotton swabs

**PREPARATION**

1. Put on gloves
2. Turn on the biohazard cabinet and clean it. Put sterile drape on the working area.
3. Put all tools in the biohazard cabinet. Wipe everything with VirkonS before introducing them in the biohazard cabinet.
4. Turn on the heating mat and Germinator.
5. Sterilize the instruments using the Germinator and lay them out.
6. Prepare the injection fluids.
7. Administer preoperative analgesia as per institutional guidelines.

**SURGERY**

1. Anesthetize the animal using isoflurane.
2. Cover the eyes with Duratears.
3. On the animal preparation side (not on top of the sterile drape in the cabinet), shave the area above and around the 4^th^ mammary gland using a razor blade. Remove hairs.
4. Move the mouse to the (sterile) surgical area within the biohazard cabinet, place it on its back.
5. Change gloves, then wet your gloved hands with VirkonS, wipe off using a tissue, spray 80% ethanol on them and let air-dry.
6. Cover the mouse with a sterile cover sheet or sterile tissue with a hole cut in it, leaving the surgical area visible.
7. Clean the surgical area three times with betadine, and then with ethanol 80%.
8. Hold the skin over the midline between both 4^th^ mammary glands using the Iris curved forceps and make a ~10 mm vertical midline incision through the skin using the Iris 24mm scissors.
9. Make 2 horizontal incisions in the skin: 1 incision anterior from the 4th mammary gland, 1 incision posterior of the 4th mammary gland. Be careful not to disrupt the mammary fat pad or the underlying peritoneum.
10. Detach the skin and mammary gland from the peritoneum using blunt dissection/gentle pulling with curved forceps.
11. Place the imaging box (preheated on the heat pad) next to the mouse and clean it with VirkonS 1% solution and ethanol 80%.
12. Transfer the mouse into the imaging box on top of a custom metal inlay with a hole that has a coverglass superglued to it. Open the skin flap, and place the 4th mammary gland on the glass, next to the rest of the body. Put the head of the mouse inside the inhalation nozzle of the imaging box.
13. Put sterile gauze soaked in preheated PBS (37°C) on top of the skin flap to keep it hydrated.
14. Use parafilm taped to the box inlay to fix the mammary gland in its position.
15. If necessary, put gentle pressure on the mammary gland with a rolled-up tissue and tape to improve the visibility of the mammary ducts. Secure the mouse with parafilm and tape.
16. Place the imaging box in the microscope.
17. Provide appropriate nutrition during imaging (see guide 4, Mouse nutrition during IVM).
18. Set up the imaging, and keep monitoring the mouse by checking the breathing, and adjust the % of isoflurane if needed.
19. After completion of IVM, gently remove the tape and parafilm from the mouse and the skin flap, turn the mouse on its dorsal side on sterile drape in the safety cabinet.
20. Close the skin with continuous stitching. Close the extremities of the suture with reef knots.
21. Let the mouse recover while the cage is partially placed on the heating mat for ~1 hour.
22. Switch off the isoflurane station, clean the imaging box and inlay with VirkonS and ethanol 80%.
23. Provide postoperative analgesia in consultation with your institute’s veterinarian.

**REMARKS**

- Follow aseptic practice during surgeries.
- Review the analgesia strategy with your local veterinarian.
- Every time before touching a mouse, disinfect hands with VirkonS, wipe off using a tissue, disinfect with ethanol 80% and let evaporate, so that the mouse is as minimally as possible exposed to VirkonS.
- By using a tissue (or other surgery cover) to cover the mouse the surgical area stays cleaner, especially during suturing, when the thread often goes through the mouse' hair.
- Preferably work in couples.
- Leave the anaesthetized mouse on the heating pad as much as possible.
- All surgical instruments are sterile and sterilized between animals using a glass bead sterilizer (Germinator).

**4) Mouse nutrition during IVM**

**HEALTH AND SAFETY**

Beware of needlestick injuries, scratch and bite wounds when handling the mouse. Take caution when working with 80% ethanol and VirkonS; avoid contact with skin and eyes, and avoid inhalation.

**REQUIRED MATERIALS**

- Flexible silicon tubing
- 10 ml syringe
- Infuse solution (Nutriflex® or sterile PBS)
- 80% ethanol
- Flexible needle for subcutaneous use
- Infuse system (optional)

For short-term imaging (<3 hours)

1. Inject the mouse subcutaneous with 200-300 ul of sterile PBS for hydration.

For long-term imaging (>3 hours)

1. For long-term imaging, the mouse needs to be infused with nutrients. We routinely use Nutriflex® special 70/240 (arrives in 2 components).
2. Prepare infuse Nutriflex® mix as follows in the flow hood:
   1. For 10 ml:

6.875 ml MilliQ

1.56 ml of component A

1.56 ml of component B

1. Place the needle subcutaneously, usually in the neck of the mouse and secure it with tape.
2. For manual delivery:
   1. Inject 50 µl of infuse solution every 30 minutes.
3. For continuous delivery via an infuse system:
   1. Clean the tubing as follows:
      1. Flush the tubing with 80% ethanol (use a 10 ml syringe).
      2. Flush the tubing with sterile PBS as in (i).
      3. Push air through the tubing a few times (This helps you later to push the nutrient mix through the tubing. You will see better when the nutriflex solution has reached the end of the tubing).
4. Take the end of the tubing and place it through one of the holes of the imaging box (from inside to outside, leaving the needle attachment side in the box).
5. Fill a 10 ml syringe with the prepared nutrient mix, attach the end of the tubing to the syringe and push the nutrient mix through until it reaches the end of the tubing.
6. Attach the tubing to the needle and tape the tubing to the imaging box to ensure that it will not dislodge during the imaging.
7. Insert the syringe in the infuse system, and set the speed on 100 µl per hour.
