# Supplementary material for: An Intravital Microscopy Toolbox to Study Mammary Gland Dynamics from Cellular Level to Organ Scale
Source: J Mammary Gland Biol Neoplasia. 2021 May 4;26(1):9–27. doi: 10.1007/s10911-021-09487-2 (PMC8217050; doi:10.1007/s10911-021-09487-2)
Supplement: Supplementary file 2 — Supplementary file2 (DOCX 7771 KB) [file 10911_2021_9487_MOESM2_ESM.docx]

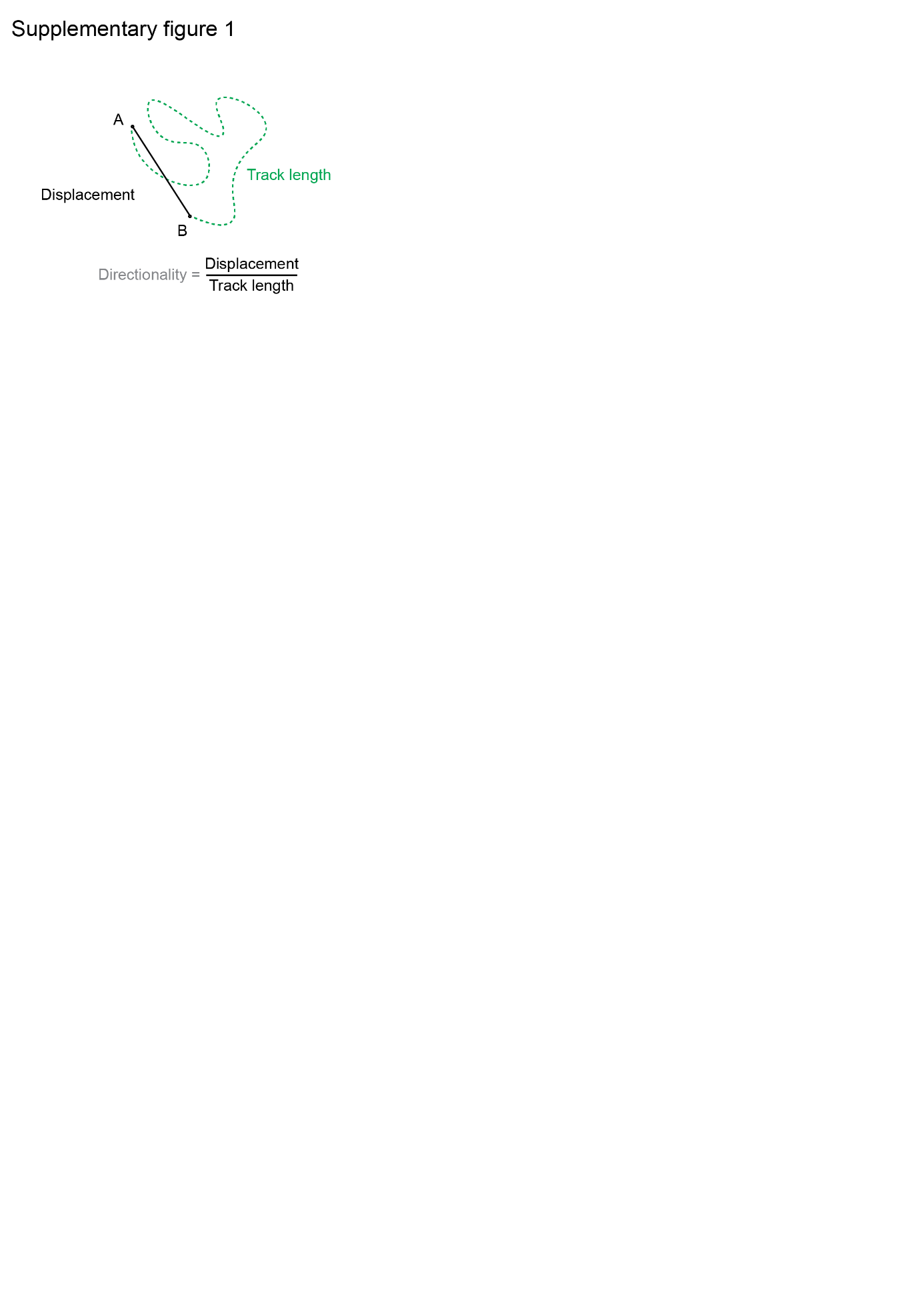
**Supplementary Figure 1.** Cartoon illustrating the different measurements obtained to describe the migratory behavior of TEB cells and ductal cells in the pubertal mammary gland. See also Figure 1.


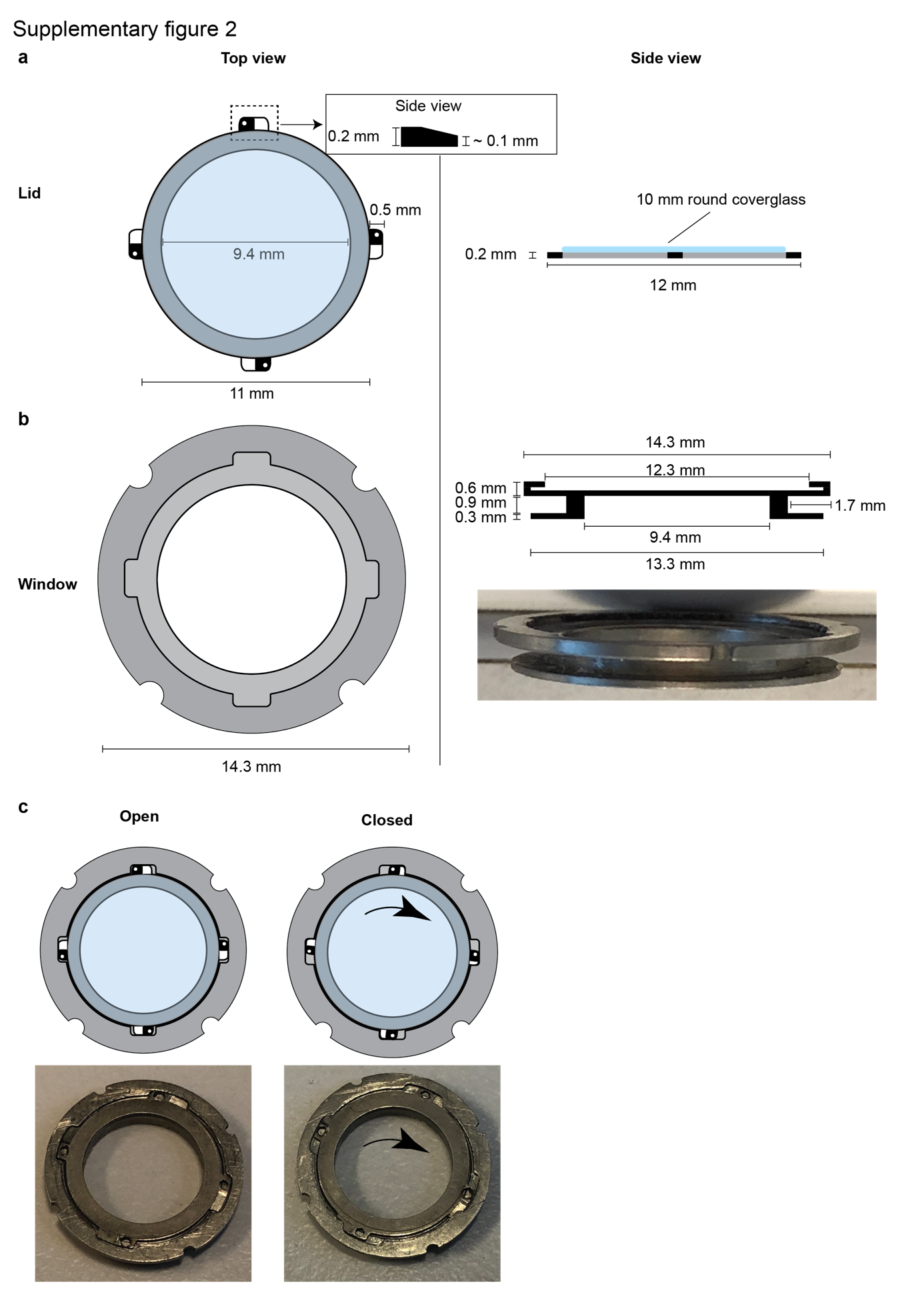
**Supplementary Figure 2: Details and design of the mammary imaging window with replaceable lid. (a)** Top view and side view of the replaceable insert of the mammary imaging window. The insert consists of a titanium ring with four slanting projections that fit within the window. A 10 mm round coverglass can be glued on top of the ring. **(b)** Top view and side view of the mammary imaging window. The mammary imaging window consists of an outer ring (larger) and inner ring (smaller, which will be inside the mouse) with a groove in between to secure the window within the skin of the mouse. The outer ring has a small groove that fits the projections of the lid. **(c)** Cartoon and pictures demonstrating the turning mechanism by which the lid can be opened and closed. The slanting projections make sure the lid is fixed inside the window.


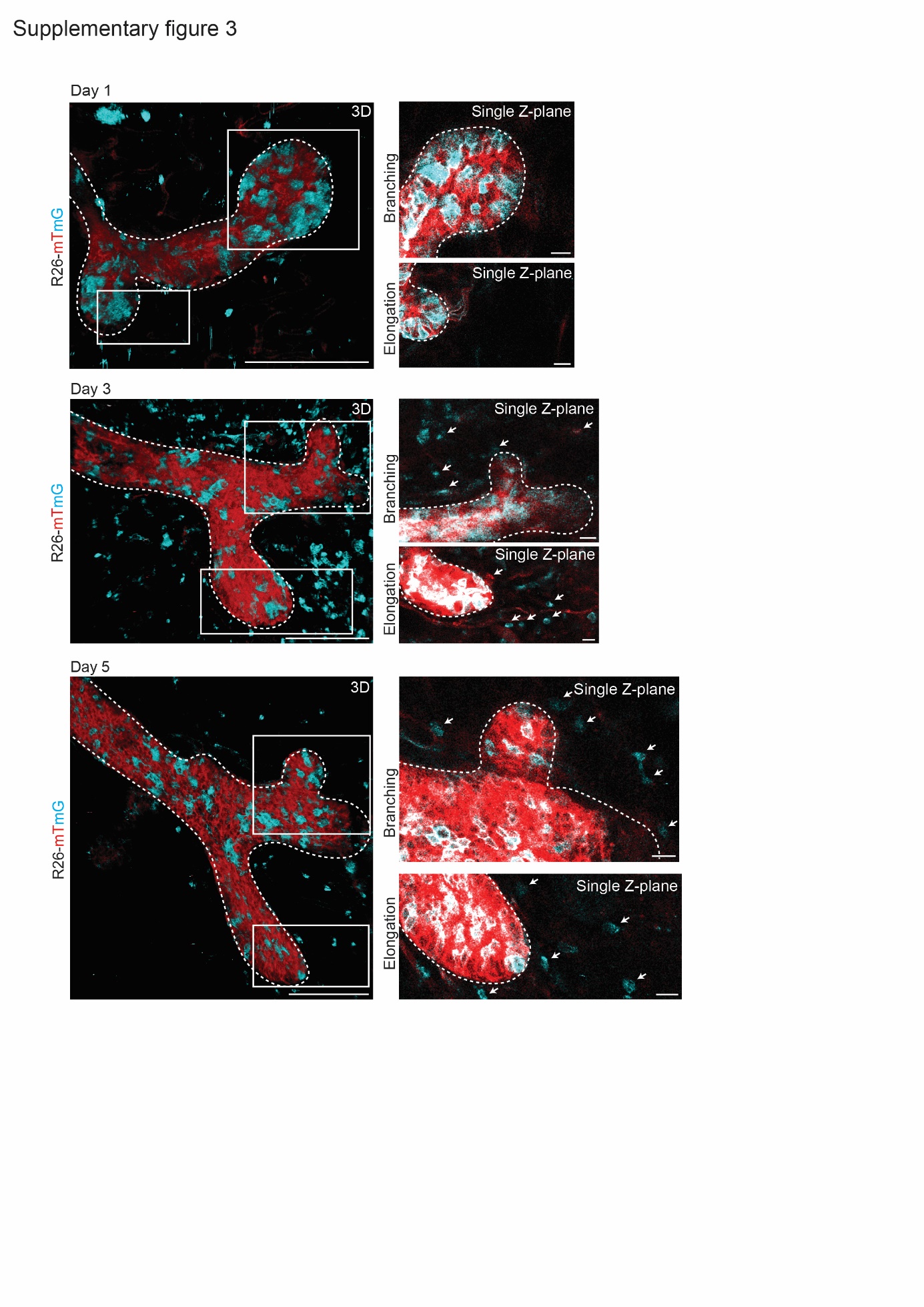


**Supplementary Figure 3: Stromal cell influx during mammary gland elongation and branching.** 3D rendering of a pubertal mammary gland structure through a MIW with replaceable lid (left, same images as shown in Figure 3d) at imaging Day 1, 3 and 5 respectively. Right panels show a single Z-plane of the branching TEB (top panels) and elongating TEB (bottom panels), demonstrating the influx of stromal or immune cells during the branching and elongation events (a subset of the incoming cells is indicated with the white arrowheads). Note that the mT and mG signal in the epithelium is much brighter compared to the stroma in the single Z-planes. To make the stroma visible we manually over-amplified the signal in the epithelium.
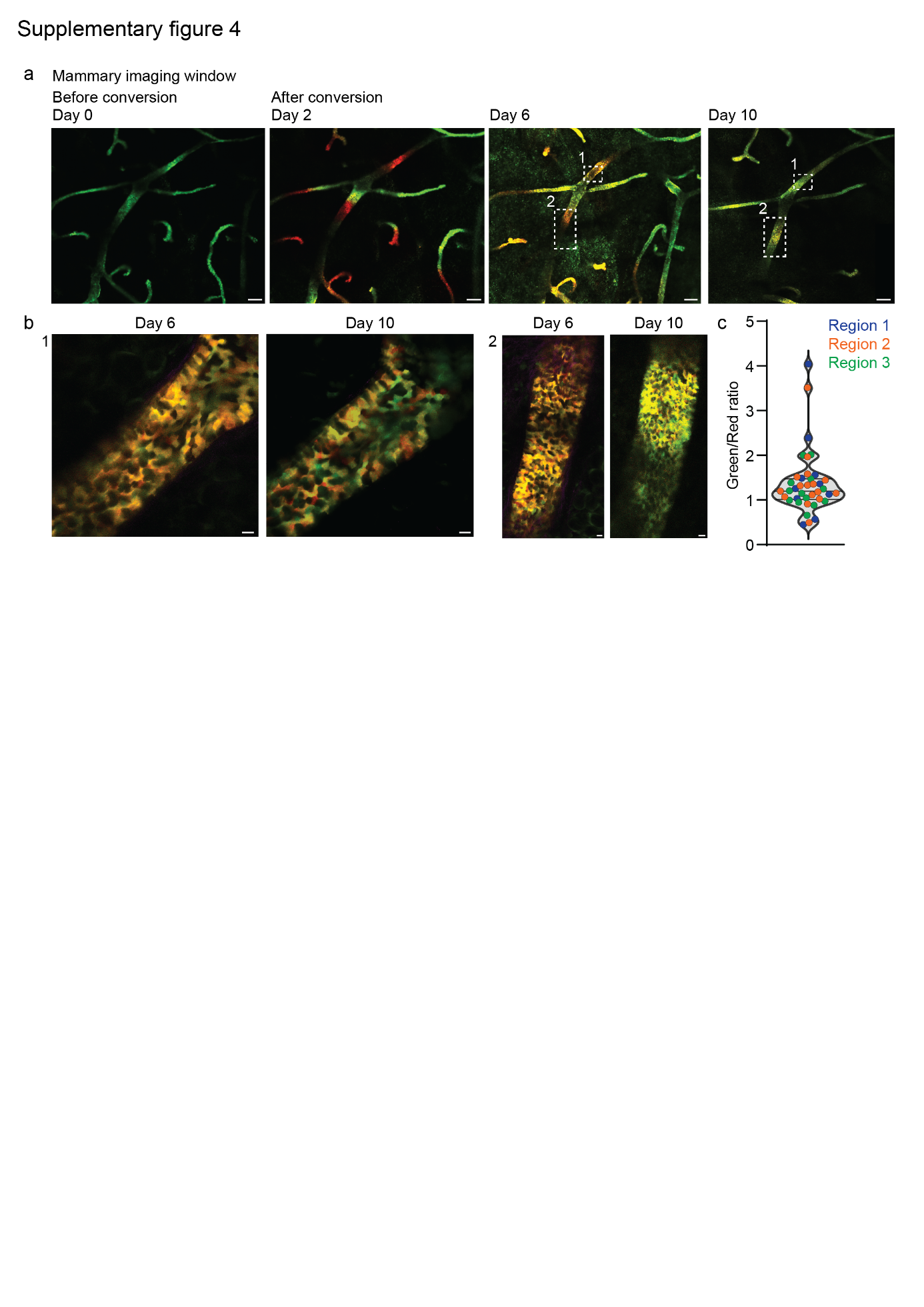
 **Supplementary Figure 4: IVM of proliferative heterogeneity during the estrous cycle in the adult mammary gland using a skin flap. (a)** Panels showing confocal images of the same ductal area (maximum intensity projection) in a KikGR mouse over a period of 10 days through a MIW, from left to right 0, 2, 6 and 10 days after exposure to 405 nm laser light. Converted areas show similar dilution of the Kikume red signal, indicating a global and equal turnover rate throughout the epithelium. Scale bar represents 100 µm. **(b)** Zoom images (single Z-plane) of different ductal areas indicated with the white boxes in panel (a) 6 and 10 days after photo-conversion, showing wide-spread proliferative heterogeneity at the cellular level. Scale bars represent 10 µm. **(c)** Graph depicts the green/Red ratio of randomly selected single cells (*n* = 48 cells) in three photo-converted areas (16 cells per area), 10 days after photo-conversion, illustrating that the majority of cells shows a similar dilution ratio. Only a minority of cells rapidly dilutes the red signal, indicative of active cycling (green/red ratio >2), or retains the red signal, indicative of quiescence (green/red ratio <0.75). Images are representative of 4 independent experiments in 4 different mice.

**
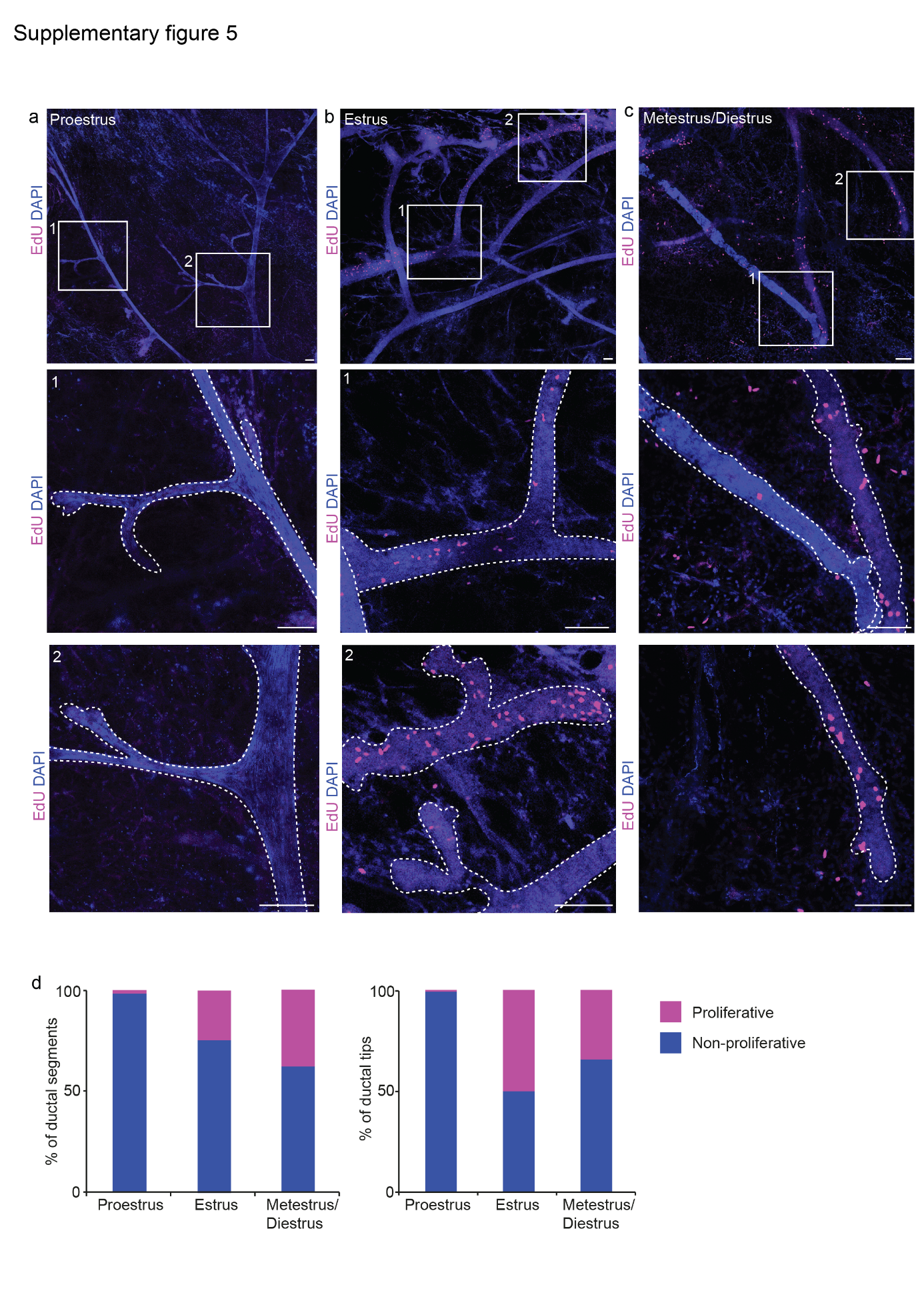
Supplementary Figure 5:** **EdU incorporation experiment shows widespread proliferation throughout the ductal tree in adult mammary gland. (a-c)** Representative whole-mount maximum intensity projections of mammary ducts 10 hours after EdU-injection during proestrus (a), estrus (b) and metestrus/diestrus (c), demonstrating scattered proliferation throughout the ductal tree during estrus and metestrus/diestrus stage. EdU is shown in magenta, nuclei (DAPI) are shown in blue. Scale bars represent 100 µm. Data is representative of 2 mice per stage. For each mouse the 4^th^ and 5^th^ mammary glands were analyzed. **(d)** Quantification of the percentage proliferative and non-proliferative ductal segments (left graph) and ductal tips (right graph) during the different stages of the estrous cycle. *n =* 60 ductal segments and 73 ductal ends (proestrus), *n =* 90 ductal segments and 82 ductal ends (estrus), and *n =* 90 ductal segments and 102 ductal ends (metestrus/diestrus).

**
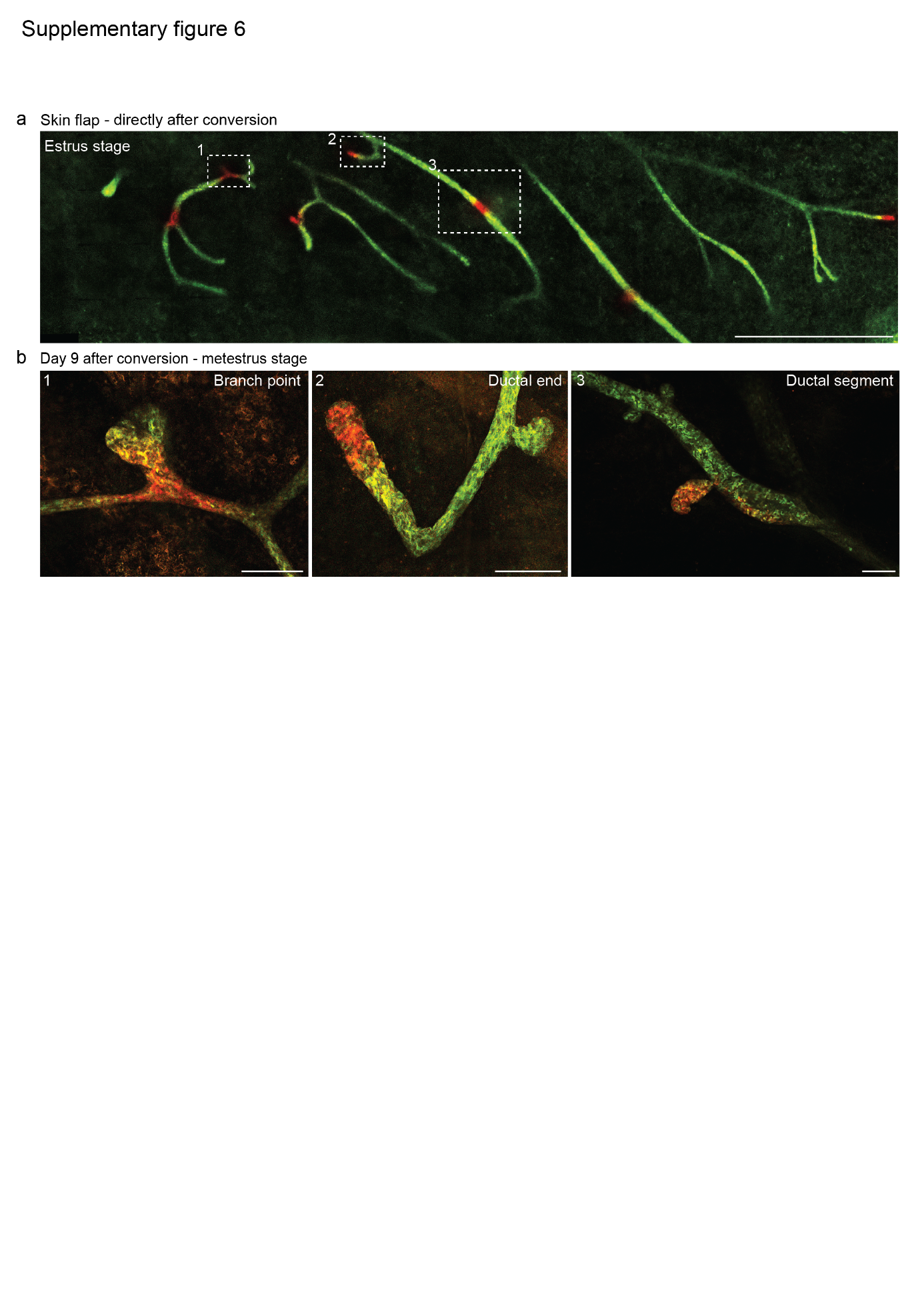
**

**Supplementary Figure 6: IVM of proliferative heterogeneity during the estrous cycle in the adult mammary gland using a skin flap. (a)** Confocal overview image (single Z-plane) of the visible area of the mammary gland using a skin flap (upper panel) directly after photo-conversion of small areas. Scale bar represents 1 mm. **(b)** Zoom images of the regions indicated with white boxes in panel (d) 9 days after photo-conversion (single Z-planes). Converted areas stayed cohesive over time, and no difference in dilution rate is observed between different regions (branch point, ductal end and ductal segment respectively) indicating similar turnover rates throughout the gland. Note that the newly formed alveolar bud in region 3 remained red (indicative of low proliferative activity), indicating that the proliferative force to create this side bud originates in the regions flanking the converted area. Scale bars represent 100 µm. Images are representative of 4 independent experiments in 4 different mice.
